# Immune response and protective efficacy of the SARS-CoV-2 recombinant spike protein vaccine S-268019-b in mice

**DOI:** 10.1038/s41598-022-25418-5

**Published:** 2022-12-02

**Authors:** Tomoyuki Homma, Noriyo Nagata, Masayuki Hashimoto, Naoko Iwata-Yoshikawa, Naomi M. Seki, Nozomi Shiwa-Sudo, Akira Ainai, Keiji Dohi, Eiji Nikaido, Akiko Mukai, Yuuta Ukai, Takayuki Nakagawa, Yusuke Shimo, Hiroki Maeda, Seiki Shirai, Miwa Aoki, Takuhiro Sonoyama, Mamoru Sato, Masataka Fumoto, Morio Nagira, Fumihisa Nakata, Takao Hashiguchi, Tadaki Suzuki, Shinya Omoto, Hideki Hasegawa

**Affiliations:** 1 Laboratory for Bio-Drug Discovery, Shionogi & Co., Ltd., 3-1-1, Futaba-cho, Toyonaka, Osaka 561-0825 Japan; 2 Department of Pathology, National Institute of Infectious Diseases, 4-7-1, Gakuen, Musashimurayama-shi, Tokyo 208-0011 Japan; 3 Laboratory for Bio-Modality Research, Shionogi & Co., Ltd., 3-1-1, Futaba-cho, Toyonaka, Osaka 561-0825 Japan; 4 UMN Pharma Inc., 7F, Tekko Building, 1-8-2, Marunouchi, Chiyoda-ku, Tokyo 100-0005 Japan; 5 Laboratory for Drug Discovery and Disease Research, Shionogi & Co., Ltd., 3-1-1, Futaba-cho, Toyonaka, Osaka 561-0825 Japan; 6 Medical Science Department, Shionogi & Co., Ltd., 8F, Nissei East Building, 3-3-16, Imabashi, Chuo-ku, Osaka 541-0032 Japan; 7 Laboratory of Medical Virology, Institute for Life and Medical Sciences, Kyoto University, 53 Shogoin Kawahara-cho, Sakyo-ku, Kyoto 606-8507 Japan; 8 Department of Virology, Faculty of Medicine, Kyushu University, 3-1-1 Maidashi, Higashi-ku, Fukuoka 812-8582 Japan; 9 Department of Pathology, National Institute of Infectious Diseases, 1-23-1, Toyama, Shinjuku-ku, Tokyo 162-8640 Japan; 10 Center for Influenza and Respiratory Virus Research, National Institute of Infectious Diseases, 4-7-1, Gakuen, Musashimurayama-shi, Tokyo 208-0011 Japan

**Keywords:** Immunology, Medical research

## Abstract

Vaccines that efficiently target severe acute respiratory syndrome coronavirus 2 (SARS-CoV-2), the etiological agent for coronavirus disease (COVID-19), are the best means for controlling viral spread. This study evaluated the efficacy of the COVID-19 vaccine S-268019-b, which comprises the recombinant full-length SARS-CoV-2 spike protein S-910823 (antigen) and A-910823 (adjuvant). In addition to eliciting both Th1-type and Th2-type cellular immune responses, two doses of S-910823 plus A-910823 induced anti-spike protein IgG antibodies and neutralizing antibodies against SARS-CoV-2. In a SARS-CoV-2 challenge test, S-910823 plus A-910823 mitigated SARS-CoV-2 infection-induced weight loss and death and inhibited viral replication in mouse lungs. S-910823 plus A-910823 promoted cytokine and chemokine at the injection site and immune cell accumulation in the draining lymph nodes. This led to the formation of germinal centers and the induction of memory B cells, antibody-secreting cells, and memory T cells. These findings provide fundamental property of S-268019-b, especially importance of A-910823 to elicit humoral and cellular immune responses.

## Introduction

Severe acute respiratory syndrome coronavirus 2 (SARS-CoV-2) is the etiological agent for coronavirus disease (COVID-19), which was first identified in Wuhan (Hubei Province, China) in December 2019. The World Health Organization (WHO) declared the SARS-CoV-2 outbreak as a Public Health Emergency of International Concern on January 30, 2020, and as a pandemic on March 11, 2020. SARS-CoV-2 infections and COVID-19 remain a global health problem, which as of April 24, 2022, has caused over 6 million deaths worldwide with over 500 million confirmed cases^1^. The staggering impact this virus has had, and continues to have, on the world population demonstrates the vital need for means to intervene and reduce its spread and disease severity. Developing vaccines that effectively target SARS-CoV-2 is the best approach for controlling the viral spread and curbing the pandemic^2^.

Coronaviruses are enveloped, single-stranded RNA viruses that can infect a wide range of hosts. However, only six coronaviruses with the ability to infect humans had been identified before the emergence of SARS-CoV-2^3,4^. Of these six coronaviruses, four cause only mild symptoms (OC43, 229E, NL63, and HKU1), while two are novel viruses that are major public health concerns as they are associated with high infectivity and lethality in humans[severe acute respiratory syndrome coronavirus (SARS-CoV) and Middle East respiratory syndrome coronavirus (MERS-CoV)]^3^. SARS-CoV-2 is the third novel highly pathogenic coronavirus to be identified to infect humans. The SARS-CoV-2 genome shares approximately 80% and 50% sequence identities with the SARS-CoV and MERS-CoV genomes, respectively^2^. Four structural proteins, including the spike protein, which is a membrane protein, constitute the SARS-CoV-2 virion^2,5^. The spike protein directly interacts with the cellular receptor angiotensin-converting enzyme 2 (ACE2) on the host cell through its receptor-binding domain (RBD)^6^. The biological functions and characteristics of the spike protein makes it an obvious vaccine target.

During the early period of spread, the rate of changes in SARS-CoV-2 was slower than that in other RNA viruses, including influenza virus and human immunodeficiency virus (HIV)^7^. However, the Omicron variant of SARS-CoV-2 has recently emerged, which exhibits increased infectivity and transmissibility. Increased pathogenic traits of the Omicron variant can be attributed in part to immune evasion, which is associated with decreased virus-neutralizing activity of the serum antibodies among vaccinated individuals^8^. While two doses of mRNA vaccine are insufficient against the Omicron variant, homologous and heterologous booster doses after two doses of mRNA vaccine elicit neutralizing responses against Omicron variant^8–10^. This suggests that a multi-dose vaccination strategy involving boosting can be effective in countering Omicron and other novel variant strains of SARS-CoV-2. Therefore, there is a need to develop sustainable routine vaccinations for COVID-19, which will require a continuous supply of SARS-CoV-2 vaccines.

Vaccines using mRNA have been developed and used worldwide to combat the COVID-19 pandemic, but concerns have been raised regarding potential side effects and the need for appropriate supply chain equipment for the production, distribution, and storage of these vaccines. Gilbert and Lambe^11^ suggested the need to develop diverse vaccine modalities in addition to mRNA vaccines to address these concerns. Recombinant proteins are a well-established vaccine platform that can employ various expression systems. Additionally, recombinant protein technology is an efficient, relatively inexpensive, and widely available tool^12^, and thus, has been used in the production of various vaccine antigens, including hemagglutinin (HA) for influenza^13^, glycoprotein E for varicella-zoster^14^, hepatitis B virus surface antigen^15^, and SARS-CoV-2 spike protein^16^.

We have been developing S-268019-b, which is an adjuvanted vaccine for COVID-19 comprising the recombinant full-length SARS-CoV-2 spike protein S-910823 as the antigen. The antigen S-910823 is designed based on the SARS-CoV-2 Wuhan-Hu-1 sequence (GenBank ID: MN908947) and includes mutations in the furin cleavage site and two substituted proline residues to improve antigen stability. The S-910823 protein is produced using a rhabdovirus-free baculovirus expression system and is mixed with A-910823, a squalene-based, oil-in-water emulsion adjuvant. Previous studies suggest the Th2-dominant immune response is suspected to cause vaccine-associated disease enhancement (VDE)^17,18^. We therefore screened and selected A-910823, oil-in-water emulsion-based adjuvant, on a basis of the Th1/2 balance and enhancement of immunogenicity. S-268019-b (S-910823 plus A-910823) was demonstrated to be immunogenic in both humans^19^ and non-human primates^20^.

The development of diverse vaccines for SARS-CoV-2 is essential now that the virus has become a human pathogen with global distribution. The current study aimed to further evaluate S-268019-b, which is under clinical development. The immunogenicity of S-268019-b in mice was assessed by examining its ability to induce both humoral and cell-mediated responses. The protective efficacy of S-268019-b and the adjuvanticity of A-910823 were also evaluated. The findings of this study will aid in the development of safe and effective vaccines for controlling the COVID-19 pandemic and help provide general protection against SARS-CoV-2 and its variants.

## Results

### Immunogenicity of S-910823 plus A-910823 in mice following intramuscular dosing

**Figure 1:**
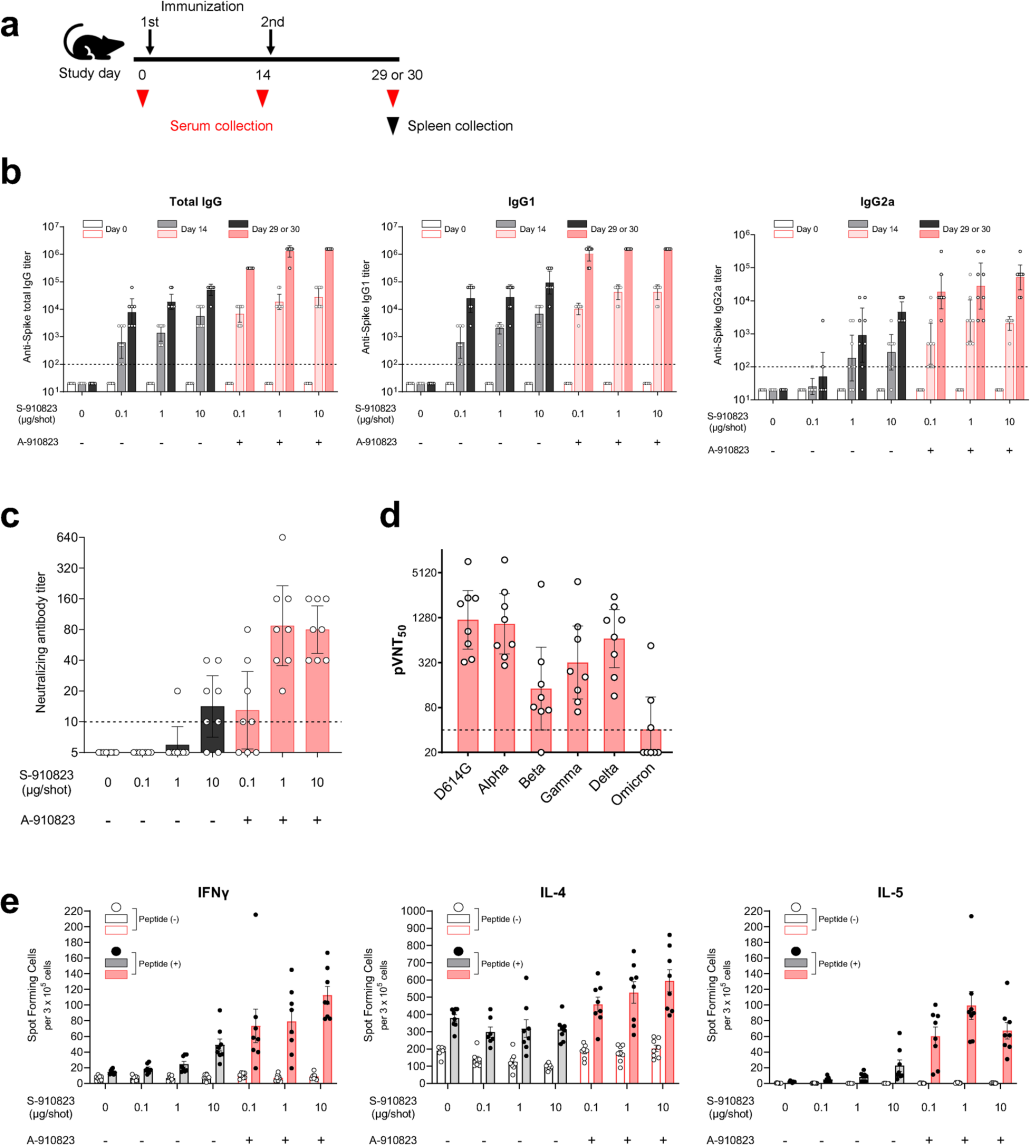
Immunogenicity of S-910823 plus A-910823 in mice after intramuscular dosing. (**a**) Study schedule schematic. (**b**) Antigen-specific total IgG, IgG1, and IgG2a titers in the serum were measured using enzyme-linked immunosorbent assays. Each bar represents the geometric mean titer (error bars indicate a 95% confidence interval). Each circle represents the titer in an individual mouse. Dotted lines indicate the lower limit of detection. (**c**) Serum titers of neutralizing antibodies against severe acute respiratory syndrome coronavirus 2 (SARS-CoV-2). Each bar represents the geometric mean titer (error bars indicate a 95% confidence interval). Each circle represents the neutralizing antibody titer in individual mice. The dotted line indicates the lower limit of detection. (**d**) Serum titers of neutralizing antibodies against SARS-CoV-2 spike pseudotyped lentivirus in mice administered 1 μg of S-268019-b. Each bar represents the geometric mean titer (error bars indicate a 95% confidence interval). Each circle represents the neutralizing antibody titer in individual mice. The dotted line indicates the lower limit of detection. (**e**) The number of cytokine-secreting cells in the spleen of immunized mice was determined using enzyme-linked immunospot analysis. Each bar represents mean spot-forming cells (SFCs) (error bars indicate standard error of mean). The open and solid bars indicate non-stimulated splenocytes and splenocytes stimulated with SARS-CoV-2 spike-protein peptides, respectively. The gray bars represent mice administered S-910823 without A-910823, while the rose bars represent mice administered S-268019-b. The open and solid circles represent the results of individual mice.

In preliminary experiments, 1 µg of S-910823 plus A-910823 increased the anti-spike IgG and neutralizing antibody titer to a peak level in mice. To confirm the dose-dependency of S-910823 and necessity of A-910823, immunogenicity experiments were conducted in the range of 0.1 – 10 µg of S-910823 with or without 50% (v/v) A-910823 at a 14 d interval (Fig. 1a). The anti-spike total IgG, IgG1, and IgG2a titers were measured on the day before the first injection (Day 0), the day before the second injection (Day 14), and 14 d or 15 d post-second injection (Day 29 or Day 30, respectively). The first dose of S-910823 plus A-910823 induced the production of anti-spike IgG, IgG1, and IgG2a antibodies, which increased further after administration of the second dose (Fig. 1b). The geometric mean titers (GMTs) of total IgG, IgG1, and IgG2a in mice administered S-910823 plus A-910823 were higher than those in mice administered S-910823 alone. This indicated that A-910823 enhanced the immunogenicity of S-910823. While the GMTs of total IgG, IgG1, and IgG2a tended to be S-910823 dose-dependent, the levels of total IgG and IgG1 almost plateaued upon administration of 1 μg of S-910823 plus A-910823 with GMTs of 1,277,758 and 1,562,500, respectively. Meanwhile, A-910823 enhanced the levels of both IgG1 and IgG2a after the second dosing. Specifically, the GMTs of IgG1 and IgG2a in mice administered 1 μg of S-910823 alone (27,951 and 914, respectively) were lower than those in mice administered 1 μg of S-910823 plus A-910823 (1,562,500 and 27,951, respectively). As IgG1 and IgG2a isotypes are markers of Th1 and Th2 lymphocytes, respectively^21–23^, these findings suggest that the adjuvant A-910823 enhanced both Th1 and Th2 responses in mice.

To assess the functional quality of the antigen-specific antibodies induced by S-268019-b, the levels of neutralizing antibodies were evaluated on Day 29 or Day 30. Consistent with the results of the anti-spike IgG titers, S-910823 plus A-910823 dose-dependently increased the titers of neutralizing antibodies against the SARS-CoV-2 Wuhan strain WK-521 (Fig. 1c). The GMTs of neutralizing antibodies in mice administered vehicle, 0.1 μg of S-910823 alone, or 1 μg of S-910823 alone were below the limit of detection. Meanwhile, the GMTs of neutralizing antibodies plateaued at 87 in mice administered ≥ 1 μg of S-910823 plus A-910823. The levels of neutralizing antibodies against pseudotyped viruses, including variant strains, were also examined (Fig. 1d). The susceptibility of the spike protein variant D614G to serum antibody is similar to that of the Wuhan strain^24^. We found the GMT of neutralizing antibodies elicited by 1 μg of S-910823 plus A-910823 against the Alpha variant (1,058) was similar to that of neutralizing antibodies elicited against the D614G strain (1200). However, the GMTs of neutralizing antibodies elicited by 1 μg of S-910823 plus A-910823 against the Beta, Gamma, Delta, and Omicron variants were 143, 321, 670, and 41, respectively, which were 8.4-fold, 3.7-fold, 1.8-fold, and 29-fold lower, respectively, than the GMT of neutralizing antibodies elicited against D614G.

Next, the effect of S-268019-b on the induction of cellular immune responses and Th1/Th2 polarity was evaluated by measuring the production of Th1 (IFN-γ) and Th2 (IL-4 and IL-5) cytokines from splenocytes collected on Day 29 or Day 30 using enzyme-linked immunospot (ELISPOT) assays (Fig. 1e). Stimulation of splenocytes with spike-protein overlapping peptides increased the mean number of spot-forming cells (SFCs) producing each cytokine in the S-910823-administered group. The numbers of antigen-specific SFCs producing IFN-γ (Th1), IL-4 (Th2), and IL-5 (Th2) in the S-910823 plus A-910823 groups tended to be higher than those in the S-910823 alone groups. This indicates that S-910823 elicited both Th1 and Th2 responses, which were further enhanced upon co-administration of the adjuvant A-910823.

### Protection of S-910823 plus A-910823 vaccinated mice against SARS-CoV-2 challenge

**Figure 2:**
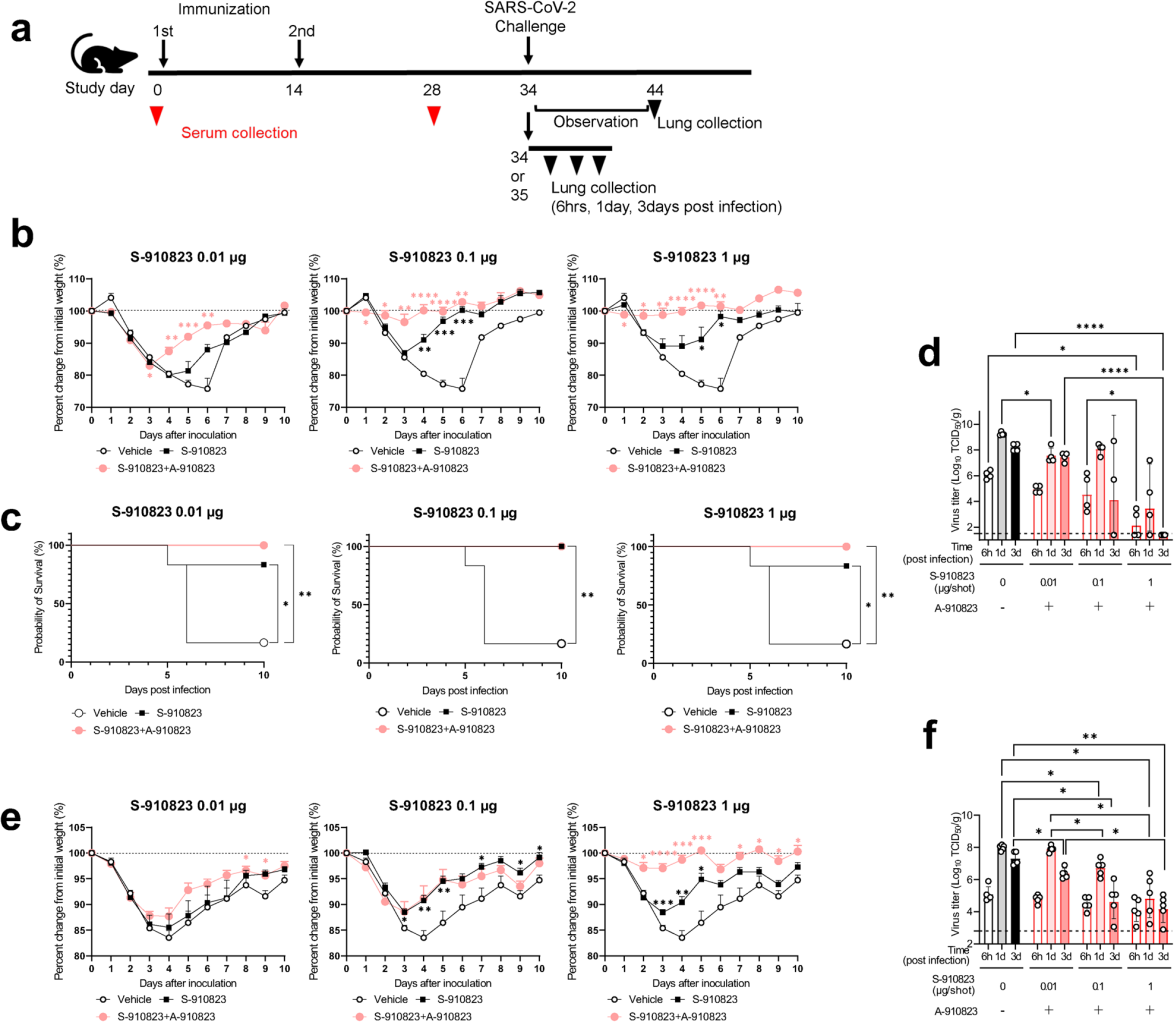
Protective efficacy of S-910823 plus A-910823 in a mouse infection model. (**a**) Study schedule schematic. On 20 d or 21 d post-second immunization, female mice were intranasally infected with severe acute respiratory syndrome coronavirus 2 (SARS-CoV-2). Bodyweight and mortality were monitored daily. The lungs were harvested at 6 h, 1 d, 3 d, and 9 d or 10 d post-infection. (**b**) and (**e**) Bodyweight changes in mice after infection with QHmusX (b, n = 6) or TY-8–612 (e, n = 8). Bodyweight of only surviving mice was measured. Each point represents the mean bodyweight of the particular group of mice (error bars indicate standard error of mean). Statistical significance was measured using Dunnett’s multiple comparison test (*P < 0.05, **P < 0.01, ***P < 0.001, and ****P < 0.0001). (**c**) Survival curves of mice after infection with QHmusX. Statistical significance was measured using the log-rank test (*P < 0.05 and **P < 0.005). (**d**) and (**f**) Infectious virus titers in mouse lungs at 6 h, 1 d, and 3 d post-infection with SARS-CoV-2 QHmusX (d, n = 4) and SARS-CoV-2 TY-8–612 (f, n = 5). The titers are expressed as median tissue culture infective dose (TCID_50_)/g of tissue in VeroE6/TMPRSS2 cells. The circles represent the titers in individual mice. The bars represent the geometric mean titers (error bars indicate standard deviation). The dotted lines represent the lower limit of detection. Statistical significance was determined using Tukey’s multiple comparison test (*P < 0.05, **P < 0.01, and ****P < 0.0001).

To evaluate the protection conferred by S-268019-b, mice administered S-910823 plus A-910823 were challenged with SARS-CoV-2 viruses, including a variant strain. Mice administered S-268019-b were intranasally infected with the mouse-passaged D614G strain QHmusX^25^ or the Beta variant TY-8–612 (Fig. 2a). S-910823 plus A-910823 vaccination promoted increased IgG titers (Supplementary Fig. S1). The QHmusX strain, which exhibits increased virulence in mice^25^, was used to evaluate the protection conferred by S-910823 plus A-910823 against SARS-CoV-2 infection. To evaluate disease severity, bodyweight changes and survival rates after infection were measured for use as disease indices for up to day 10 post-infection (Fig. 2b, c)^26^. All mice in the vehicle-administered group had lost weight at 2 d post-infection. Of the six mice in the vehicle-administered group, five had not survived to 6 d post-infection. In contrast, almost all mice in the S-910823-administered group survived during the entire observation period, except for one mouse each in the groups receiving 0.01 or 1 μg of S-910823. The average bodyweight of S-910823-administered mice at 3 d or 4 d post-infection was lower than that on Day 0, the day before infection, but this was followed by rebound increases in bodyweight. Mice administered with 0.1 or 1 μg of S-910823 plus A-910823 did not exhibit a drop in bodyweight.

The titers of infectious virus in the lung homogenates of mice infected with the Wuhan strain were determined using the Behren-Karber method and expressed as median tissue culture infectious dose (TCID_50_)/g of tissue. The viral titers in mice administered 1 μg of S-910823 plus A-910823 were lower than those in other groups at all tested time points (6 h, 1 d, and 3 d post-infection; Fig. 2d). Notably, the viral titers at 3 d post-infection in mice administered 1 μg of S-910823 plus A-910823 were below the lower limit of detection. In contrast, the viral titers at 3 d post-infection were above the limit of detection in the vehicle-administered mice and mice administered 0.01 or 0.1 μg of S-910823 plus A-910823. The cytokine and chemokine levels in the lung homogenates of mice administered 1 μg of S-910823 plus A-910823 were lower than those in mice of the other groups at all tested time points (Supplementary Fig. S2). Furthermore, the levels of Th1-associated (IL-2), Th2-associated (IL-4 and IL-5), and Th17-associated (IL-17) cytokines and chemokines in mice administered 1 μg of S-910823 plus A-910823 were lower than those in mice administered 0.01 or 0.1 μg of S-910823 (Supplementary Fig. S2).

The widely adopted mRNA vaccines exhibit the least neutralizing activity against the Beta variant of SARS-CoV-2 among the variants of concern that evolved before the “Omicron era”^27^. In the current mouse experiment, neutralizing activity induced by S-910823 plus A-910823 against the Beta variant was lesser than that against the D614G strain (Fig. 1d). Therefore, the effect of S-910823 plus A-910823 vaccination on bodyweight of mice infected with the Beta variant was next examined. An obvious decrease in bodyweight was observed in the vehicle-administered group, but none of the mice died. The lack of mortality may have been a result of the Beta variant being less pathogenic in mice compared to that of the mouse-adapted QHmusX strain. The bodyweight loss in mice administered 0.1 or 1 µg of S-910823 plus A-910823 was lower than that in mice administered vehicle. However, mice administered 1 µg of S-910823 plus A-910823 did not exhibit bodyweight loss after infection (Fig. 2e). Furthermore, lung histopathology scores in mice administered 1 μg of S-910823 plus A-910823 were improved compared with those in mice administered vehicle or those in the other vaccine groups, as demonstrated by the absence of alveolar or vascular necrosis, and weak cellular inflammation (Supplementary Fig. S3). Next, the titers of infectious virus in the lungs of mice infected with the Beta variant were evaluated at 6 h, 1 d, and 3 d post-infection. S-910823 plus A-910823 dose-dependently decreased the viral titers in the lungs with the viral titers being 3-log_10_ lower in mice administered 1 μg of S-910823 plus A-910823 compared with those administered vehicles at 1 d and 3 d post-infection (Fig. 2f).

### Eosinophilic histopathological analysis of S-910823 plus A-910823 immunized mice post-SARS-CoV-2 challenge

**Figure 3:**
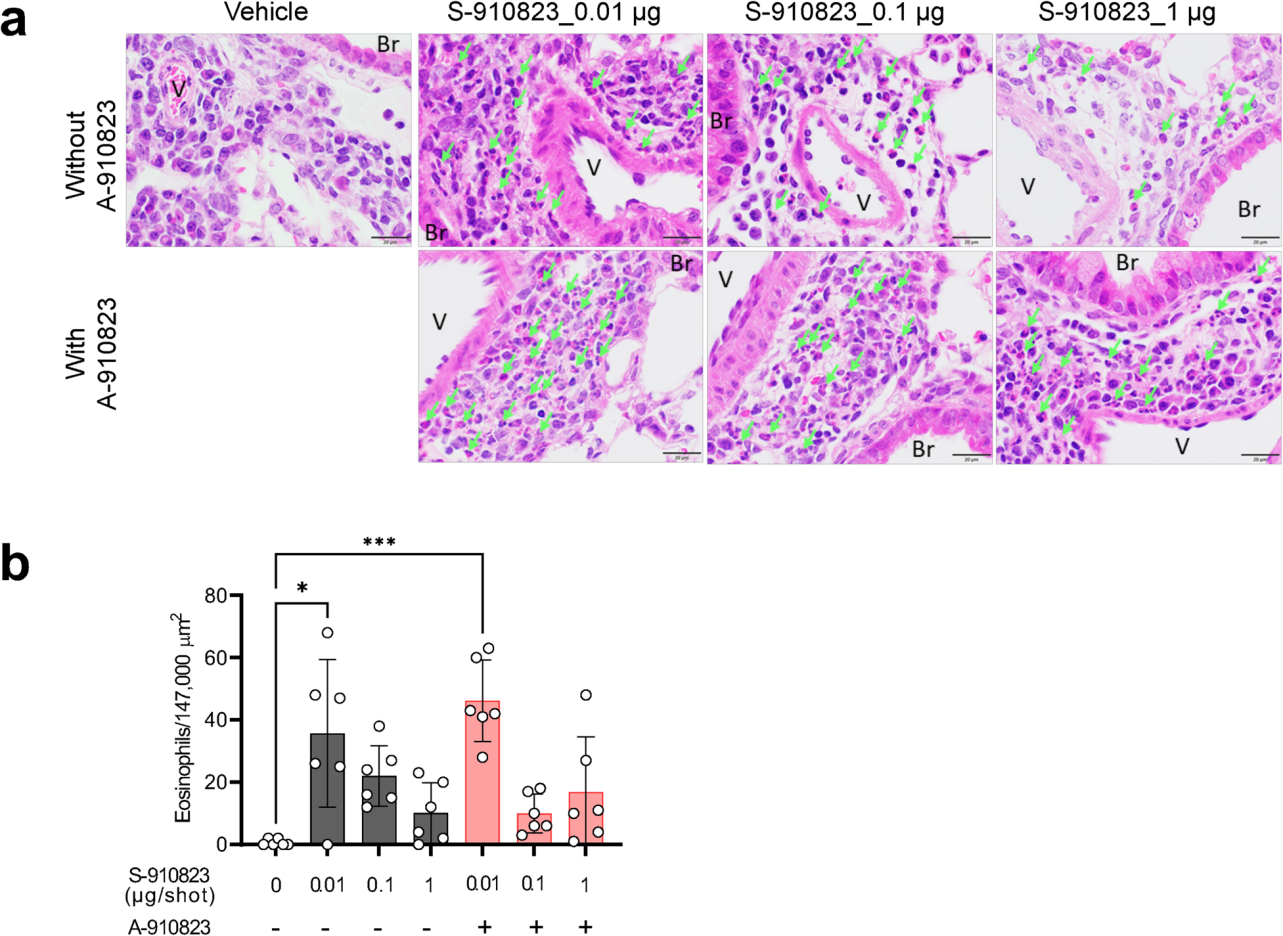
Eosinophilic infiltration in the lungs of vaccinated mice after challenge infection with severe acute respiratory syndrome coronavirus 2 (SARS-CoV-2). Lung specimens were collected at the humane endpoint or 10 d post-infection. The study schedule is described in detail in Fig. 2. (**a**) Representative histopathological findings from mice with the highest levels of eosinophilic infiltration. Infiltration was detected using eosinophilic staining with a combined eosinophil­mast cell (C.E.M.) staining kit. Scale bar = 100 μm. Green arrows indicate representative eosinophils. Br, bronchus; V, blood vessel. (**b**) Number of eosinophils per lung section. The number of eosinophils in five regions with an area of 147,000 μm^2^ around the pulmonary bronchioles of each mouse was counted at 40 × magnification. Each circle represents the results of individual animals. The bar represents the mean number of eosinophils (error bars indicate standard deviation). Statistical significance was determined using a nonparametric comparison test, followed by the Kruskal–Wallis test (*P < 0.05 and ***P < 0.001).

Previous studies have reported that SARS-CoV or SARS-CoV-2 challenge infection after vaccination elicits Th2-dominant responses and induces eosinophilic infiltration owing to insufficient neutralizing antibody levels^25,28^. Hence, the lungs of vaccinated mice challenged with SARS-CoV-2 were subjected to histopathological analysis. The analysis was performed 10 d post-infection or at the humane endpoint when necessary. Representative histopathological images of lungs of immunized mice infected with QHmusX are shown in Fig. 3a. Histopathological evaluation revealed eosinophilic infiltrations in the lungs of mice administered S-910823 only or S-910823 plus A-910823 (Fig. 3a). In contrast, eosinophilic infiltration was not observed in the lungs of vehicle-administered mice. The density of eosinophilic infiltration into the lung tissue of the mice was quantified by counting the number of cells (Fig. 3b). S-910823 dose-dependently decreased the levels of eosinophilic infiltration. A-910823 did not affect the number of infiltrating eosinophils. This suggests that sufficient dosing of S- S-910823 plus A-910823 can mitigate eosinophilic infiltration and that the adjuvant A-910823 did not increase eosinophilic infiltration.

### A-910823-mediated enhancement of S-910823 immunogenicity

**Figure 4:**
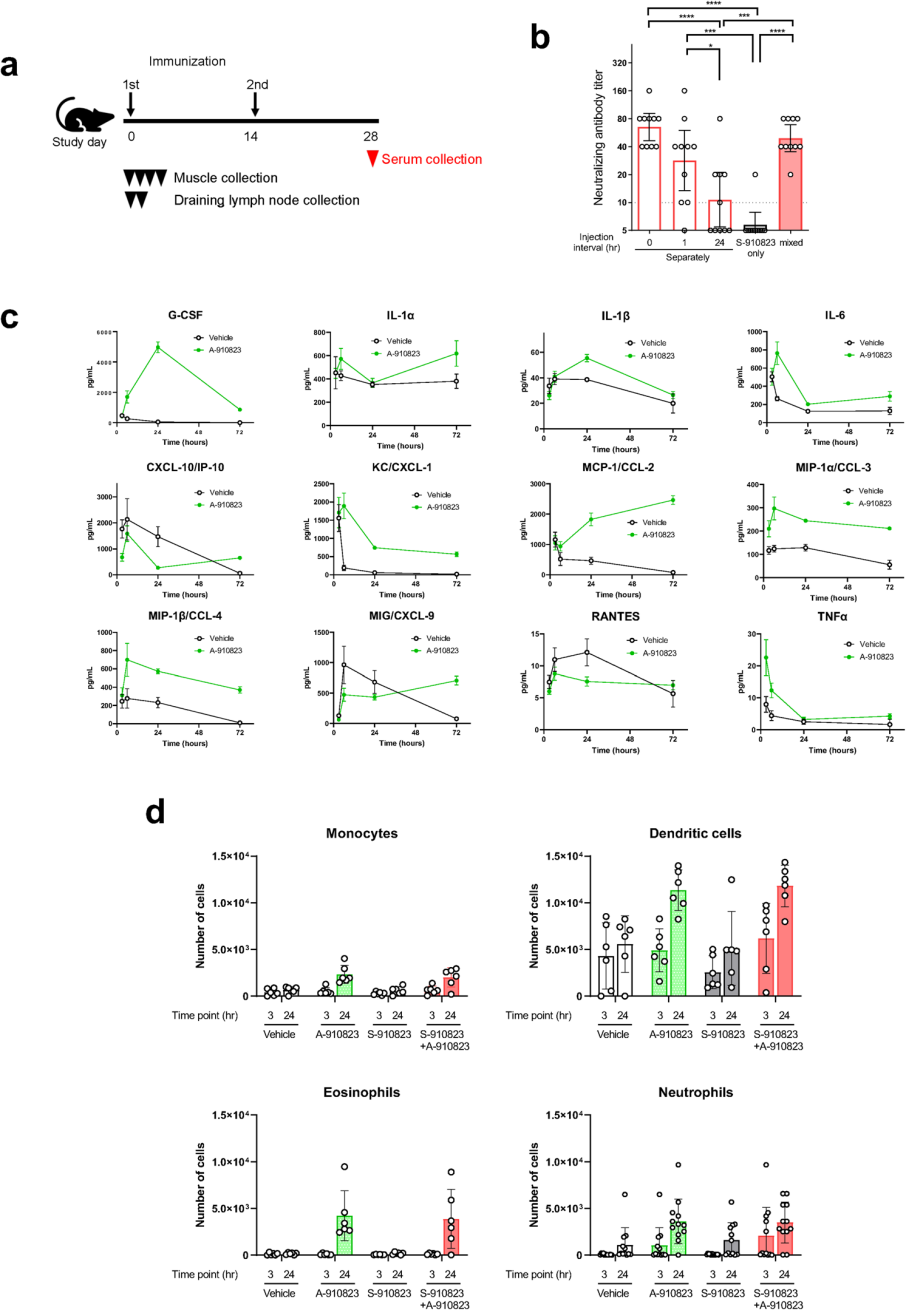
Effect of A-910823 on S-910823 immunogenicity. (**a**) Study schedule schematic. (**b**) Physical association of S-910823 with A-910823 in mice. S-910823 (1 μg) and A-910823 (100% v/v) were separately administered into mice at different injection intervals (0 h, 1 h, and 24 h) on Day 0 and Day 14 (n = 10/group). Serum titers of neutralizing antibodies against authentic SARS-CoV-2 virus were measured in mice at 2 wk post-second immunization. Each bar represents the geometric mean titer (error bars indicate a 95% confidence interval). The circles represent the results of individual animals. The dashed line indicates the lower limit of detection. Statistical significance was determined using one-way analysis of variance, followed by Tukey’s multiple comparison test (*P < 0.05, ***P < 0.001, and ****P < 0.0001). (**c**) Cytokine and chemokine concentrations in the muscle homogenate supernatant at 3 h, 6 h, 24 h, and 72 h post-administration of 50% (v/v) A-910823 or vehicle (n = 4–7 per time point). Data are represented as mean ± standard error. (**d**) Quantification of inflammatory cells in the draining lymph nodes at 3 h and 24 h post-administration of vehicle or 1 μg of S-910823 with or without A-910823 (n = 6/group). The bars represent the mean number of cells (error bars indicate standard error of mean). The circles represent the results of individual animals.

The adjuvanticity of A-910823 was essential for S-268019-b to elicit both humoral and cellular immune responses. Therefore, the mechanistic characterization of A-910823 was performed (Fig. 4a). To determine if the adjuvant activity of A-910823 involved a physical association with S-910823 after immunization, S-910823 antigen and A-910823 adjuvant were injected separately or together, and the serum neutralizing antibody titers were comparatively analyzed. The antibody titers were not significantly different between mice administered S-268019-b and those administered A-910823 separately within 1 h post-S-910823 injection (Fig. 4b). This indicated that the adjuvanticity of A-910823 was not dependent on a physical interaction with the S-910823 antigen. However, the neutralizing antibody titers in mice administered A-910823 24 h after S-910823 administration were significantly downregulated when compared with those in mice administered S-910823 plus A-910823. This may be attributed to the degradation or passive dispersion of the antigen, which may affect the immunogenicity of S-910823.

Next, a multiplex assay system was used to analyze the expression of 12 different cytokines and chemokines in the homogenates of local muscle tissues collected from the A-910823 injection site up to 72 h post-injection (Fig. 4c). A-910823 induced the production of granulocyte colony stimulating factor (G-CSF, neutrophil-mobilizing cytokine) and keratinocytes-derived chemokine (KC, neutrophil-recruiting chemokine) at 24 h and 6 h post-injection, respectively. Additionally, A-910823 induced the production of the proinflammatory cytokines IL-6 and IL-1β at 6 h and 24 h post-injection, respectively. Furthermore, A-910823 promoted the production of Monocyte chemoattractant protein-1 (MCP-1), macrophage inflammatory protein-1α (MIP-1α), and MIP1β.

To assess the innate immune cell-recruiting capacity of A-910823, the number of dendritic cells, monocytes, neutrophils, and eosinophils in the draining lymph nodes of mice was evaluated at 3 h and 24 h post-intramuscular injection of A-910823 and/or S-910823 (Fig. 4d and Supplementary Fig. S4). The absolute numbers of all cell types in the draining lymph nodes increased at 24 h post-administration with A-910823 alone or A-910823 plus S-910823. However, the number of each cell type did not increase after the administration of S-910823 alone. These results indicate that A-910823 could induce immune cell accumulation in the draining lymph nodes of the injected muscle by promoting the production of cytokines and chemokines.

### Generation of follicular helper T (Tfh) cells and germinal centers B cells (GCBs) in the draining lymph nodes of mice administered S-910823 plus A-910823

**Figure 5:**
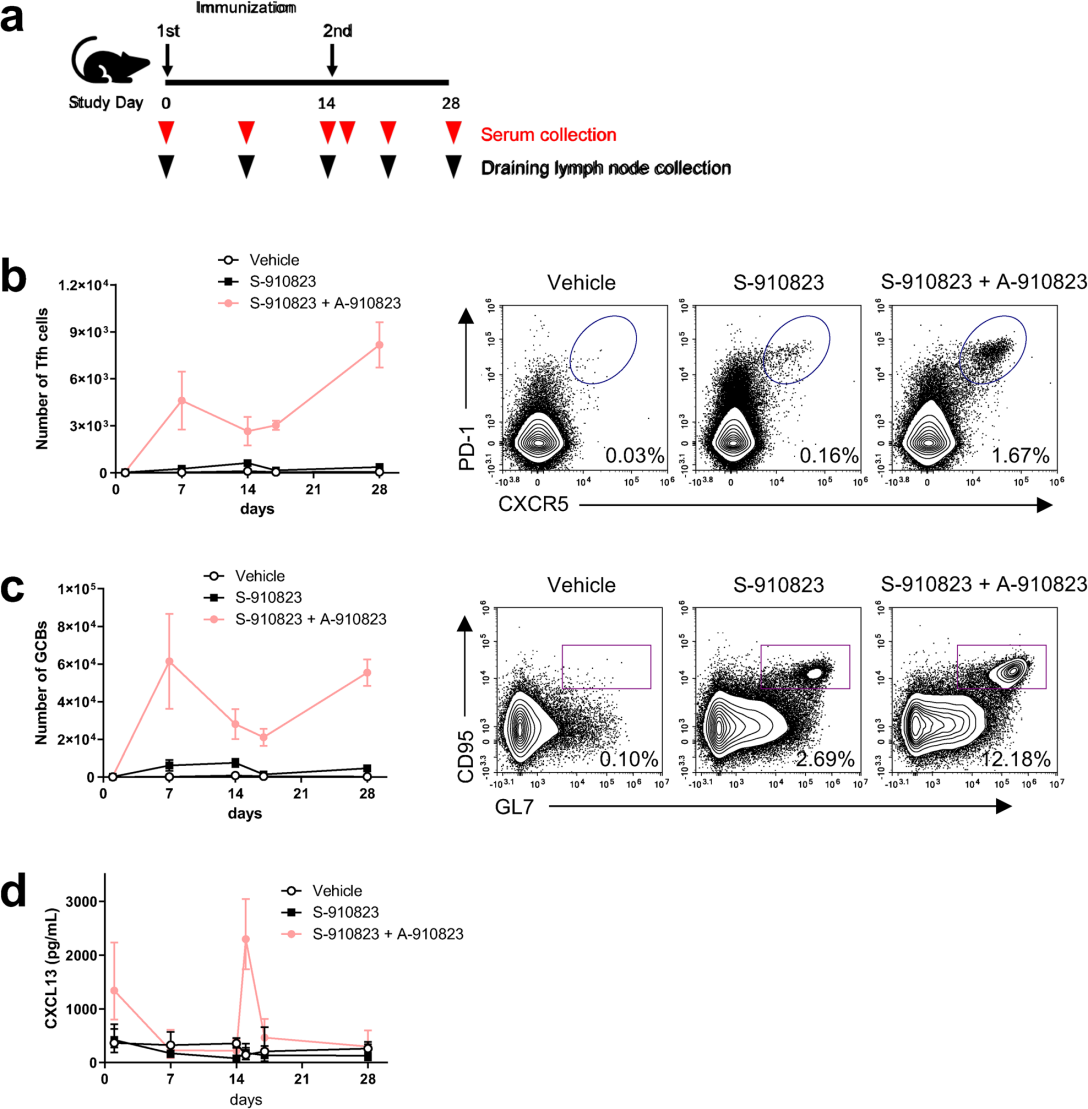
Generation of follicular helper T (Tfh) cells and germinal center B-cells (GCBs) in the draining lymph node of mice administered S-910823 plus A-910823. (**a**) Study schedule schematic. Mice were intramuscularly administered 1 μg of S-910823 with or without A-910823 on Day 0 and Day 14. Draining lymph nodes were collected from the immunized animals (n = 5/group) on days 1, 7, 14, 17, and 28 post-first administration. Serum was prepared from the blood samples collected on days 1, 7, 14, 15, 17, and 28 post-first immunization. (**b**) The number of Tfh cells and a representative contour plot. (**c**) The number of GCBs and a representative contour plot. (**d**) Serum levels of CXCL13 in immunized animals. The graph points represent mean cell number values (**b**, **c**) or mean concentration values (**d**). The error bars represent standard error of mean (**b**, **c**) or 95% confidence intervals (**d**).

Effective vaccines induce the GC reaction in draining lymph nodes. The GC response improves the affinity of the B-cell receptor (BCR) through somatic hypermutation of the BCR-encoding gene^29^. To assess the GC-inducing capacity of the A-910823-adjuvanted vaccine, the GC-related cell types, such as Tfh cells and GC B-cells (GCBs), in the draining lymph node of the vaccinated mice were quantified (Fig. 5a). The absolute number of Tfh cells and GCBs in mice administered S-910823 plus A-910823 was higher than that in mice administered with S-910823 only. The number of Tfh cells and GCBs increased at 7 d post-first injection, which increased further after the second injection (Fig. 5b, c, and Supplementary Fig. S5). Additionally, CXCL13, which is a candidate marker of GC induction in mice, macaques, and humans^30^, was transiently upregulated immediately after the administration of S-910823 plus A-910823, prior to the formation of GCBs and Tfh cells (Fig. 5d). Furthermore, administration of S-910823 plus A-910823 upregulated the expression levels of genes related to B-cell maturation, including *Aicda*, *Il-21*, *Prdm1*, *Il-4*, and *Tnfrsf17* (Supplementary Fig. S6).

### Induction of long-term immunity in mice administered S-910823 plus A-910823

**Figure 6:**
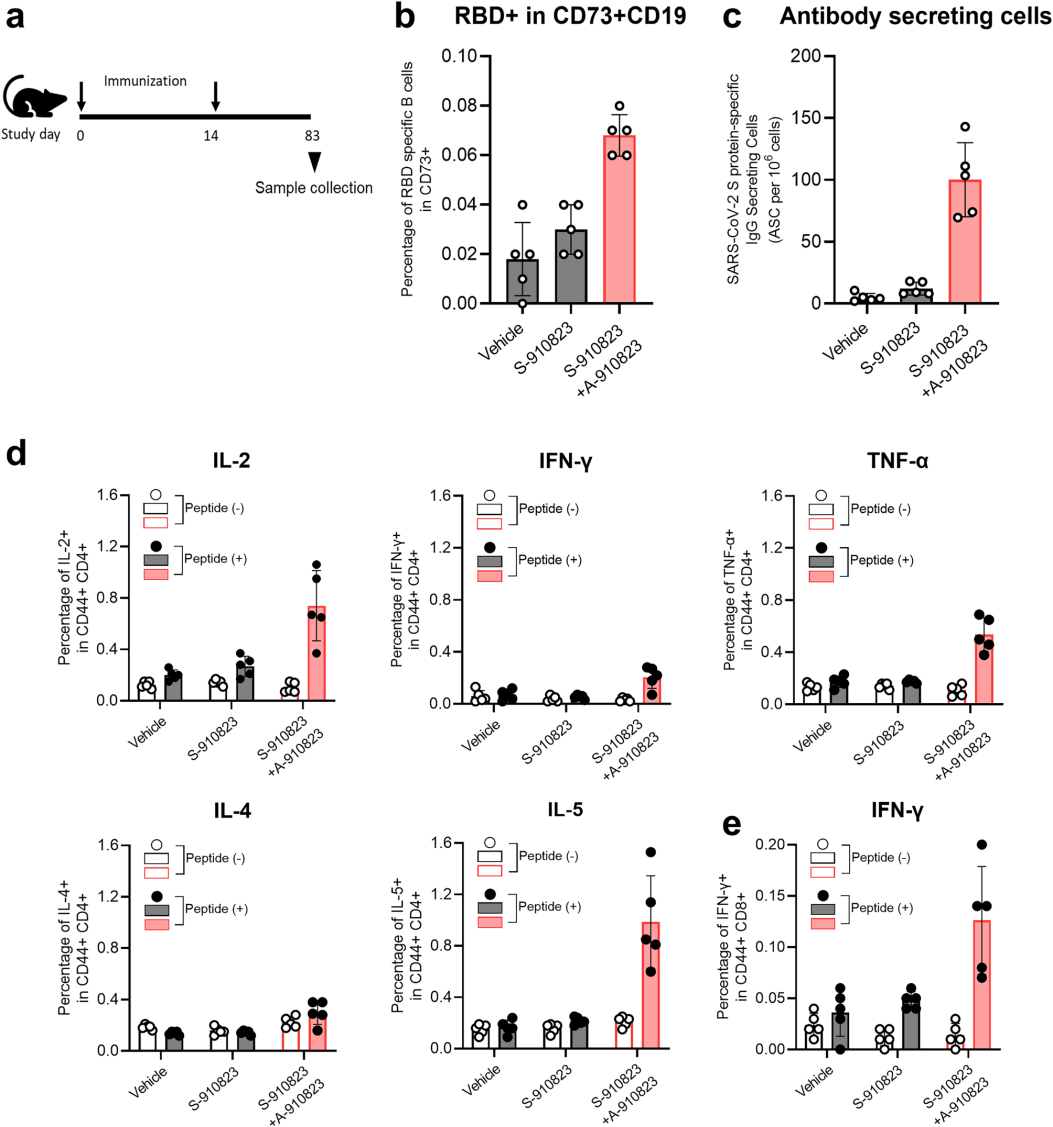
Induction of long-term immunity in mice administered S-910823 plus A-910823. (**a**) Study schedule schematic. On Day 0 and Day 14, female mice were intramuscularly administered 1 μg of S-910823 with or without A-910823 (n = 5/group). Spleens and bone marrow were collected on Day 83. (**b**) Flow cytometric analysis of the percentage of receptor-binding domain (RBD)-specific B-cells among CD73^+^CD19^+^ B-cells. Each circle represents the percentage of cells in an individual mouse. The bars represent mean values, while error bars indicate standard deviation. (**c**) Detection of vaccine-induced antibody-secreting cells (ASCs) in bone marrow. Bone marrow cells were cultured for 2.5 h in an enzyme-linked immunospot (ELISPOT) assay plate wells which had been coated with SARS-CoV-2 spike protein. Results indicate the number of antigen-specific ASCs among 10^6^ bone marrow cells. (**d**, **e**) Percentage of cytokine-producing T cells in the spleen. Splenocytes were restimulated ex vivo with or without SARS-CoV-2 spike-protein-derived peptides for 16 h. Levels of IL-2, IFN-γ, TNF-α, IL-4, and IL-5 from CD44^+^CD4^+^ T cells (**d**) and levels of IFN-γ from CD44^+^CD8^+^ T cells (**e**) were evaluated using cell-surface and intracellular staining and examined using flow cytometry. The open bars indicate non-stimulated splenocytes [peptide ( −)], while the solid bars indicate splenocytes stimulated with SARS-CoV-2 spike protein peptides [peptide ( +)]. The gray bars represent mice administered vehicle or S-910823 without adjuvant A-910823, while the rose bars represent mice administered S-910823 with A-910823. The open and solid circles represent the results of individual mice. The error bars indicate the standard deviation.

Maintenance of memory B-cells, long-lived plasma cells (LLPC)^31,32^, and memory T-cells^33^ are crucial for establishing an anamnestic response or long-lasting immunity after vaccination. To evaluate the anamnestic response, mice were intramuscularly administered with two doses of 1 μg of S-910823 with or without 50% (v/v) of A-910823 at a 14 d interval. Spleen and bone marrow samples were collected on Day 83 post-first vaccine administration (Fig. 6a). For analysis of the memory B-cell response, SARS-CoV-2 spike protein receptor-binding domain (RBD)-specific B-cells were analyzed using flow cytometry (Supplementary Fig. S7). Flow cytometric analysis revealed that the membrane expression of CD73, a known memory B-cell marker^34^, was expressed at a higher frequency on RBD-specific B-cells of mice administered S-910823 plus A-910823 compared with that on RBD-specific B-cells of mice administered S-910823 only on Day 83 (Fig. 6b). Additionally, the number of SARS-CoV-2 spike-protein-specific antibody-secreting cells (ASCs), which are precursors of LLPCs, was higher in the bone marrow of mice administered S-910823 plus A-910823 than that in mice administered vehicle or S-910823 alone (Fig. 6c and Supplementary Fig. S8). To evaluate S-910823-specific T-cells, the splenocytes of mice were restimulated ex vivo with SARS-CoV-2-derived peptides on Day 83. As CD44 is a marker for effector-memory T-cells^35^, the cytokines produced by CD44^+^CD4^+^ T cells or CD44^+^CD8^+^ T cells of the restimulated splenocytes were detected using flow cytometry (Supplementary Fig. S9). As shown in Fig. 6d, the levels of IL-2, IFN-γ, TNF-α, and IL-5 produced by CD44^+^CD4^+^ T cells in mice administered S-910823 plus A-910823 were higher than those in mice administered S-910823 alone on Day 83 (Fig. 6d). This indicated that both Th1 and Th2 responses induced by S-910823 plus A-910823 remained detectable on Day 83 post-first vaccine administration. Additionally, the levels of IFN-γ produced by CD44^+^CD8^+^ T cells in mice administered S-910823 plus A-910823 were higher than those in mice administered vehicle or S-910823 alone (Fig. 6e). These data suggest that antigen-specific memory B-cells, LLPCs, and memory T-cells induced by S-910823 plus A-910823 were sustained for more than two months after the second immunization.

## Discussion

The global impact of SARS-CoV-2 infections, which continue to be a major health concern, has revealed the critical need for developing preventative and therapeutic interventions. In this study, the efficacy of S-268019-b, i.e., S-910823 (antigen) plus A-910823 (adjuvant), a novel COVID-19 vaccine currently under clinical development, was evaluated in mice. S-910823 plus A-910823 exhibited potent immunogenicity in mice as evidenced by the induction of anti-spike-protein IgG antibodies and the neutralization of SARS-CoV-2. The neutralizing activity of S-910823 plus A-910823 against Beta and Omicron variants was lower than that against the Wuhan strain and other variants. These immunogenic trends in mice were consistent with those observed in humans^19^ and non-human primates^20^. Therefore, a detailed analysis in this study will provide useful information regarding S-268019-b.

The development of vaccine-associated disease enhancement (VDE) is a potential complication in the successful development of vaccines against viruses and may hinder the safety of SARS-CoV-2 vaccines^17^. The major factors contributing to the development of VDE are insufficient neutralizing antibody levels or production of non-neutralizing antibodies, and a skewed Th2 response^18,36^. A Th2-dominant immune response is thought to mediate VDE by promoting the release of IL-4, IL-5, IL-13, and eosinophil chemoattractants, which leads to eosinophil infiltration and proinflammatory cytokine production^18,25,36^. However, Th2 activation is also essential to enhance antibody production^37^, and therefore, the risk of VDE should be evaluated based on both the Th2 response and any effects it may have on the infection model. In the current study, S-910823 plus A-910823 elicited both Th1 and Th2 immune responses. Furthermore, S-910823 plus A-910823 did not exacerbate the infection in mice, and actually protected the mice against SARS-CoV-2 infection-induced weight loss and mortality and inhibited viral replication in mouse lungs. This suggests that the risk of VDE caused by S-268019-b vaccination is low. Currently, COVID-19 vaccine-induced VDE is of less concern in humans than SARS-CoV-2 infection and the observation of COVID-19 as VDE has not been clinically reported, yet.

Adjuvants are critical vaccine components that enhance specific immune responses. However, an adjuvant can potentially induce undesirable immune responses, which is also a vaccine safety concern. For instance, the adjuvant alum has been shown to exacerbate immunopathological reactions in a mouse infection model^38^. In the current study, S-910823 combined with adjuvant A-910823 dose-dependently decreased eosinophilic infiltration into the lungs of vaccinated mice, which was comparable to the eosinophilic infiltration observed in mice administered the S-910823 antigen only. This suggests a low risk of VDE caused by the A-910823 adjuvant.

Animal models are essential for fully evaluating candidate vaccines. However, host factors are key determinants of COVID-19 disease severity and progression in both humans^39^ and mice^40,41^ and must be considered. Mice have been successfully used for studies on vaccines for various viruses, including SARS-CoV-2 and other coronaviruses, and mouse infection models have been advanced with the use of mouse-adapted virus strains^25,40,41^. The protective efficacy of S-910823 plus A-910823 was evaluated in the current study in a mouse infection model using a Beta strain of SARS-CoV-2 (TY-8–612). The vaccination of S-910823 plus A-910823 inhibited viral replication and prevented bodyweight loss. This efficacy was corroborated by the neutralizing activity against pseudotyped Beta strains, which was lower than that against the D614G strain, but still maintained some activity. The benefits of using mice as a model system for evaluating the efficacy of vaccines against SAR-CoV-2 in preventing COVID-19 include well-defined immunological and genetic factors, an extensive foundation of previous work, the availability of experimental reagents, and the relative ease of use and low expense as an in vivo system. However, recent efforts to establish a mouse infection model using the Omicron variant have not been successful^42^.

Since the A-910823 adjuvant worked separately from that of S-910823, A-910823 may exert effects independent of S-910823. Thus, A-910823 itself may induce innate immune responses at the site of injection. Consistent with this hypothesis, A-910823 induced several cytokines and chemokines, especially IL-6, suggesting an antibody response^43^. The induction of chemokines, such as MCP-1 and MIP1-α, can lead to the recruitment of monocytes and granulocytes into draining lymph nodes. CXCL13 induction suggests the promotion of GC formation in the draining lymph node^30^, which contributes to the induction of potent antibody responses during SARS-CoV-2 infection or after vaccination^44^. GCBs and Tfh cells interact to promote B-cell maturation and their differentiation into memory B-cells and plasma cells within the GC. To induce Tfh cells through vaccination, monocyte-derived dendritic cells must be induced after vaccination^45^. Importantly, A-910823 effectively induced the recruitment of monocytes and promoted Tfh-cell differentiation. Finally, the adjuvanticity of A-910823 induced effective GCB maturation, which subsequently induced Tfh cells and promoted the maintenance of plasma cells, memory B-cells and memory T-cells. Thus, an A-910823-adjuvanted vaccine can sequentially induce innate and adaptive immunity, which leads to humoral and cellular immune responses against the SARS-CoV-2 spike protein.

The long-term immunity and broader neutralizing activity are significant features for future strategy of COVID-19 vaccination, including boosting administration. The longevity of immunity elicited by approved COVID-19 vaccines were investigated and neutralizing effect of serum collected from vaccine recipients lasted 90 days or longer^46^. The antigen-specific T-cell and B-cell with memory phenotype also induced by mRNA vaccination^47,48^. In this study, we revealed that antigen-specific memory B-cell, LLPCs, and memory T-cell were induced by S-910823 plus A-910823 in mice. In addition, the boosting capability of S-910823 plus A-910823 after SARS-CoV-2 infection was confirmed, which displayed the breadth expansion of neutralizing activity against Omicron variant (Supplementary Fig. S10). On the one hand, booster immunization of S-268019-b after mRNA vaccination elicited more sustained antibody response than mRNA booster^49^. Intriguingly, this boosting also induced stronger antibody response to Omicron RBD compared to mRNA booster, although lower magnitudes than Wuhan RBD^49^. The accumulating evidence suggests usefulness of S-268019-b after immunization by vaccination or SARS-CoV-2 infection in terms of long-term immunity and broader neutralizing activity. We are currently undertaking a detailed investigation on maintenance of memory B and T cells in clinical trials.

The current study had several limitations. As noted, the pathogenesis of SARS-CoV-2 infections and vaccine efficacy differ between humans and mice. Therefore, the findings of this study should be validated in clinical studies or additional experiments using other animal species, including non-human primates.

This was the first study to evaluate the immunogenicity of S-268019-b in mice. The findings consistently indicate that S-268019-b was safe and effective in the in vivo model system. Furthermore, these findings provide fundamental property of S-268019-b, especially importance of A-910823 to elicit humoral and cellular immune responses, in effort to provide safe and efficient tools for combating the COVID-19 pandemic. This is not only currently important but will also benefit the management of SARS-CoV-2 and its variants and other novel coronaviruses that may emerge in the future.

## Methods

### Cell lines, SARS-CoV-2, and pseudoviruses

VeroE6/TMPRSS2 cells^50^ were obtained from the Japanese Collection of Research Bioresources Cell Bank (Osaka Japan). The cells were cultured in Dulbecco’s Modified Eagle Medium (DMEM; Thermo Fisher Scientific, Waltham, MA, USA) supplemented with 10% heat-inactivated fetal bovine serum (FBS; Corning, Corning, NY, USA) and 1 mg/mL gentamicin (Genticin; Thermo Fisher Scientific). The SARS-CoV-2 virus hCoV-19/Japan/TY-WK-521/2020, Pango lineage A (WK-521) was obtained from the Department of Virology III, National Institute of Infectious Diseases (NIID), Japan. The QHmusX strain was previously isolated^25^. The SARS-CoV-2 Beta variant (hCoV-19/Japan/TY-8–612/2021) was obtained from NIID, Japan. The SARS-CoV-2 MA-P10 strain (mouse-adapted WK-521) was provided from the Division of Molecular Pathobiology, International Institute for Zoonosis Control, Hokkaido University, Sapporo, Japan^51^. The viruses were propagated in VeroE6/TMPRSS2 cells to prepare virus stock solutions and cryopreserved at − 80 °C. Viral infectivity titers were determined in VeroE6/TMPRSS2 cells using tissue culture infectious dose (TCID_50_) assays. The pseudoviruses were prepared as previously described^20^. Briefly, the plasmids pGAG, pREV, and pCDH (System Biosciences, Palo Alto, CA, USA), and pGL4 (Promega, Madison, WI, USA) were prepared and used to generate HIV-based pseudotyped lentiviruses. SARS-CoV-2 S genes from the strains B.1 (GISAID: EPI_ISL_529135), B.1.1.7 (GISAID: EPI_ISL_601443), B.1.351 (GISAID: EPI_ISL_768642), P.1 (GISAID: EPI_ISL_792680), B.1.617.2 (GISAID: EPI_ISL_1914591), and BA.1 (GISAID: EPI_ISL_6640917) were codon-optimized for human cells and cloned into eukaryotic expression plasmids to generate the envelope recombinant plasmids pSPIKE_D614G, pSPIKE_Alpha, pSPIKE_Beta, pSPIKE_Gamma, pSPIKE_Delta, and pSPIKE_Omicron, respectively. To generate the D614G, Alpha, Beta, Gamma, Delta, and Omicron spike protein pseudotyped lentiviral Luc2 reporter viruses, Lenti-X 293 T cells (Takara Bio Inc., Shiga, Japan) were co-transfected with pGAG, pREV, pCDH-CAG-Luc2, and the relevant pSPIKE plasmid using FuGENE HD (Promega). The pseudovirus copy number in the viral supernatants was measured using a Lenti-X™ qRT-PCR Titration Kit (Takara Bio Inc.), according to the manufacturer’s instructions.

### Vaccine (S-268019-b), antigen (S-910823), and adjuvant (A-910823)

The S-268019-b vaccine (Shionogi & Co., Ltd., Osaka, Japan) was prepared in-house and comprised the indicated concentrations of antigen S-910823 (Shionogi & Co., Ltd.) with or without the oil-in-water emulsion-based adjuvant A-910823 (Shionogi & Co., Ltd.). The recombinant S-910823 protein was expressed using a baculovirus expression vector system as previously described^20^. Briefly, the recombinant spike protein S-910823 was expressed in baculovirus-infected cells and extracted with nonionic detergent, followed by filtration of cell extracts using depth filter. S-910823 was purified using affinity chromatography, hydrophobic interaction chromatography, and anion exchange chromatography. Tangential flow filtration was used for protein concentration and buffer exchange. The purified S-910823 was analyzed by reduced SDS-PAGE followed by Coomassie Brilliant Blue staining (Supplementary Fig. S11). The indicated concentrations of S-910823 protein were prepared in 0.02% PS20 buffer as vehicle (15 mM phosphate buffer, 150 mM sodium chloride, 0.02% w/v polysorbate 20, pH 7.5) with or without 50% (v/v) A-910823. The mean particle size of A-910823 ranged 100—200 nm, and the polydispersity was under 0.20.

### Animals for immunogenicity and challenge infection studies

All animal experiments in this article were conducted in accordance with the ARRIVE guidelines. Female BALB/cAJcl mice were obtained from CLEA Japan, Inc. (Tokyo, Japan) and dosed with used at 8 or 9 wk of age for vaccine dosing and adjuvant analysis studies. For virus challenge studies, female BALB/cCrSlc mice were obtained from Japan SLC (Hamamatsu, Shizuoka, Japan) or Charles River Japan (Atsugi, Kanazawa, Japan) and used at 12 wk of age. For vaccination, mice were twice intramuscularly administered 50 μL of the appropriate samples under isoflurane anesthesia at a 14 d interval (Day 1 and Day 15). For virus challenge studies, vaccinated mice were intranasally inoculated under anesthesia with the QHmusX strain (2.3 × 10^4^ TCID_50_/30 µL) or the SARS-CoV-2 Beta variant TY-8–612 (2.3 × 10^4^ TCID_50_/50 µL) at 20 d post-second vaccine injection. The infected mice were monitored for clinical signs of infection and their bodyweight measured daily for 10 d or until progression to a humane endpoint. The humane endpoint was defined as the appearance of clinically diagnostic signs of respiratory stress, including respiratory distress, and weight loss of more than 25%. To perform viral titration and cytokine expression analyses, mice were sacrificed at 6 h, 1 d, and 3 d post-inoculation, and their lungs were collected. For boosting experiment, mice were intranasally inoculated under anesthesia with MA-P10 (1 × 10^4^ TCID_50_/50 µL), and then, were immunized at 85 d post-infection. All animal studies were performed according to the Guidelines for Proper Conduct of Animal Experiments of the Science Council of Japan. The animal experiments strictly complied with animal husbandry and welfare regulations and were approved by the Committee on Experimental Animals at the NIID in Japan (approval No. 521006) and the Institutional Animal Care and Use Committee of Shionogi & Co., Ltd (approval No. S20073C).

### Serum preparation

To evaluate the immunogenicity after intramuscular dosing, small volumes of blood were obtained from each mouse under isoflurane anesthesia on the day before the first vaccine injection (Day 0) and on the day before the second vaccine injection (Day 14). Mice were sacrificed under isoflurane anesthesia and whole blood and spleen samples were obtained from each mouse 14 d (Day 29) or 15 d (Day 30) after the second vaccine injection. Due to task allocation, Day 29 and Day 30 were regarded as the same sampling points. Four mice in each group were sacrificed on Day 29, while the other mice in each group were sacrificed on Day 30. The plasma samples (Day 0 and Day14) or serum samples (Day 29 or Day 30) were prepared from the blood samples on the day of blood collection and stored at − 80 °C until use. The spleen samples were prepared for analysis on the day of collection. For the challenge infection studies, small volumes of blood were obtained from each mouse 7 d before the first vaccine administration and 14 d after the second vaccine administration. The serum samples were prepared from the blood samples and stored at − 80 °C until the assays were performed. The serum samples were used for measurement of antigen-specific IgG titers by enzyme-linked immunosorbent assay (ELISA) and for neutralizing antibody assays.

### Anti-spike protein IgG and neutralizing antibody analysis

To measure the immunogenicity after intramuscular dosing, the antigen-specific IgG titers were measured using ELISAs. A 1 μg/mL solution of SARS-CoV-2 spike protein His Tag Super Stable Trimer (ACROBiosystems, Newark, DE, USA) was used as a capture antigen in the 384-well assay plates. After washing with phosphate-buffered saline (PBS) containing 0.05% Tween 20 (PBS-T), the samples were blocked with 1% bovine serum albumin (BSA) in PBS-T. The plasma samples were fivefold serially diluted in the range of 1/100 to 1/7,812,500 with PBS-T containing 0.1% BSA (dilution buffer). The serum samples were fivefold serially diluted in the range of 1/100 to 1/4,882,812,500 with dilution buffer. The diluted samples were added to the plates in duplicate. SARS-CoV-2 (2019-nCoV) Spike Neutralizing Antibody, Mouse Mab (Sino Biological, Chesterbrook, PA, USA) was used at 4 ng/mL as a positive control for IgG and IgG1 detection. Meanwhile, SARS-CoV-2 spike RBD antibody (R&D Systems, Minneapolis, MN, USA) was used at 4 ng/mL as a positive control for IgG2 detection. Dilution buffer was used as a negative control. The samples were incubated for approximately 2 h at room temperature, followed by incubation with horseradish peroxidase (HRP)-conjugated goat anti-mouse IgG-Fc fragment (Bethyl Laboratories, Montgomery, TX, USA), HRP-conjugated goat anti-mouse IgG1 (Bethyl Laboratories), or HRP-conjugated goat anti-mouse IgG2a antibodies (Bethyl Laboratories) for approximately 1 h. Finally, the samples were incubated with 1-Step Ultra TMB-ELISA Substrate Solution (Thermo Fisher Scientific) at room temperature. Sulfuric acid was added to stop color development. The optical density at 450 nm of the sample was measured using an EnVision 2103 microplate reader (PerkinElmer, Waltham, MA, USA).

The neutralizing antibody assays for SARS-CoV-2 were performed for the immunogenicity intramuscular dosing studies. Heat-inactivated serum samples were two-fold serially diluted (range: 1/5 to 1/5120). Each sample was mixed with an equal volume of viral suspension (2,000 TCID_50_/mL) and incubated for approximately 1 h at room temperature for neutralization. After neutralization, 100 µL of the sample/virus mixtures were dispensed in duplicate into 96-well culture plates. The samples were then incubated with 100 µL of VeroE6/TMPRSS2 cell suspension (3 × 10^4^ cells/well) to obtain a virus titer of 100 TCID_50_/well. Only virus and cell suspension were added to virus control wells, while only cell suspension was added to cell control wells. The samples were incubated at 37 °C with 5% CO_2_ for 3 d. Cell viability was evaluated using CellTiter-Glo 2.0 (Promega, Madison, WI, USA). After removing 100 μL of supernatant from each well, 100 μL of CellTiter-Glo was added and the samples were incubated at room temperature for approximately 30 min under light-shielded conditions. Next, 100 μL of the mixture was transferred to measurement plates and the luminescence intensity was measured using an EnSpire 2300 microplate reader (PerkinElmer).

Neutralizing antibody assays for SARS-CoV-2 spike pseudotyped virus were also performed as previously described^20^. Briefly, heat-inactivated mouse serum samples were two-fold serially diluted, mixed with an equal volume of 4 × 10^7^ viral RNA copies of the D614G, Alpha, Beta, Gamma, Delta, or Omicron variant pseudotyped lentivirus, and incubated at 37 °C for 1 h. The mixture was then added to the prepared VeroE6/TMPRSS2 cells. At 48 h post-transduction, the cells were lysed and subjected to luciferase assays using a ONE-Glo Luciferase Assay System (Promega). Neutralization was quantified based on the reduction of luciferase gene expression. The serum dilution at which the relative light units were decreased by 50% compared with that of the virus control wells was calculated as the 50% pseudotyped virus neutralization titer (pVNT_50_) using XLfit, version 5.3.1.3 software (IDBS, Woking, UK).

### Virus titration

Mouse lung homogenates (10% w/v) were prepared in low-glucose DMEM (Sigma-Aldrich, Burlington, MA, USA) supplemented with 2% FBS, 100 IU/mL penicillin G, 100 μg/mL streptomycin, and 1.25 µg/mL amphotericin B. The homogenates were centrifuged at 650 × *g* for 20 min and the supernatants were collected and inoculated onto VeroE6/TMPRSS2 cells. The cells were incubated with the supernatant for 5 d and examined for cytopathic effects. The viral infectivity titers were calculated using the Behrens–Kärber method and expressed as TCID_50_/mL in VeroE6/TMPRSS2 cells.

### Evaluation of antigen-specific cytokine production in spleens

The spleen samples were dissociated into cell suspensions using a gentleMACS Dissociator (Miltenyi Biotec, Bergisch Gladbach, Germany). The red blood cells were lysed with 1X lysing buffer (BD Biosciences, Franklin Lakes, NJ, USA) and the splenocytes were suspended in 13 mL of Roswell Park Memorial Institute (RPMI)-1640 medium (Nacalai Tesque, Kyoto, Japan) supplemented with 1% FBS. An aliquot (100 μL) of the splenocyte suspension was mixed with 1 μL of 100-fold diluted 4′,6-diamidino-2-phenylindole solution to stain the dead cells. The number of live lymphocytes in each sample was then counted using a Novocyte Advanteon Flow Cytometer (Agilent Technologies, Santa Clara, CA, USA). The supernatant was removed from the remaining splenocyte samples and the cell number in each suspension adjusted to 3 × 10^6^ cells/mL using complete culture medium. Antigen-specific cytokine production was measured using ELISPOT assays, which were performed using ELISpot^PLUS^ kits for mouse IFN-γ, IL-4, and IL-5 (Mabtech, Cincinnati, OH, USA), following the manufacturer’s instructions with slight modifications. Briefly, ELISPOT plates were washed with PBS and blocked with complete culture medium for at least 30 min at room temperature. Spike protein overlapping peptides PepTivator SARS-CoV-2 Prot_S (Miltenyi Biotec), PepTivator® SARS-CoV-2 Prot_S1 (Miltenyi Biotec), and PepTivator® SARS-CoV-2 Prot_S + (Miltenyi Biotec) were solubilized in distilled water and diluted with complete culture medium at a concentration of 180 pmol/mL for each peptide. After removing the blocking solution, 100 µL of peptide solution was added to the ELISPOT plates. Distilled water in complete culture medium served as a peptide negative ( −) control. Next, 100 µL of splenocyte suspension was added to each well. The assay was performed in triplicate for each sample. The plates were incubated at room temperature for approximately 30 min, followed by incubation at 37 °C with 5% CO_2_ overnight. After washing five times with PBS, the samples were incubated with 100 µL of detection antibody solution for at least 2 h. The samples were washed again five times with PBS and incubated with 100 µL of diluted alkaline phosphatase (ALP)-conjugated streptavidin solution for at least 1 h. Finally, the sample was incubated with 100 µL of the chromogen 5-bromo-4-chloro-3-indolyl phosphate (BCIP)/nitro blue tetrazolium (NBT), which was used as a chromogen. The color development was stopped by washing the sample with tap water. The spots were counted using an immunospot S6 UNIVERSAL-V ELISPOT reader (Cellular Technology Limited, Beachwood, OH, USA). The mean number of spots in the triplicate wells was calculated and regarded as the number of SFCs for each sample. The mean SFC value was calculated for each group. The antigen-specific cytokine production was evaluated by comparing SFC values of peptide ( +) samples with those of peptide ( −) control wells.

### Cytokine production analysis in tissue homogenates

Homogenized lung tissue samples (10% w/v) prepared from mice infected with QHmusX were diluted 1:1 in cell extraction buffer consisting of 10 mM Tris (pH 7.4), 100 mM NaCl, 1 mM ethylenediaminetetraacetic acid, 1 mM ethylene glycol-bis(β-aminoethyl ether)-N,N,N′,N′-tetraacetic acid, 1 mM NaF, 20 mM Na_4_P_2_O_7_, 2 mM Na_3_VO_4_, 1% Triton X-100, 10% glycerol, 0.1% sodium dodecyl sulfate, and 0.5% deoxycholate (BioSource International, Camarillo, CA, USA). The samples were incubated for 10 min on ice with vortexing, irradiated for 10 min with UV-C light to inactivate the infectious virus, and examined in a biosafety level 2 laboratory. Cytokine and chemokine levels were measured using a commercial mouse cytokine-chemokine magnetic bead panel 96-well plate assay kit (MILLIPLEX MAP Mouse Cytokine/Chemokine Magnetic Bead Panel-Immunology Multiplex Assay; Merck Millipore, Burlington, MA, USA), which detects 21 different cytokines and chemokines (eotaxin, IFN‐γ, IL‐1α, IL‐1β, IL‐2, IL‐4, IL‐5, IL‐6, IL‐10, IL‐12 (p40), IL‐12 (p70), IL‐13, IL‐17, IP‐10, KC, MCP‐1, MIP‐1α, GM-CSF, MIG, RANTES, and TNF‐α). The absorbance of the samples was measured using a Luminex 200 instrument equipped with xPONENT software (Thermo Fisher Scientific), following the manufacturer’s instructions.

The thigh muscles of mice administered A-910823 were collected in PBS and homogenized using TissueRuptor II (QIAGEN, Hilden, Germany). The homogenized samples were centrifuged at 10,000 × *g* for 15 min. The G-CSF, IL-1α, IL-1β, IL-6, IP-10, KC, MCP-1, MIP-1α, MIP-1β, MIG, RANTES, and TNF-α levels in the homogenate supernatant were measured using the Luminex 200 xPONENT system.

### Lung histopathology

The lungs were harvested from euthanized infected mice. The tissue was fixed in 10% buffered formalin, sectioned, and stained with hematoxylin and eosin. Eosinophils were identified using Astra Blue/Vital New Red staining with a combined eosinophil­mast cell stain (C.E.M. Stain Kit; Diagnostic Biosystems, Pleasanton, CA, USA). The peribronchiolar regions in five 147,000 μm^2^ sections were assessed using light microscopy. Images were captured using a DP71 digital camera and analyzed using cellSens software (Olympus, Tokyo, Japan). The numbers of eosinophils in the lungs of each mouse were counted and averaged as previously described^52^. Pathological evaluation was performed according to Owen and colleagues^53^ with modifications. Briefly, histopathological evaluation was performed by two pathologists to determine alveolar epithelial degeneration or necrosis, bronchial or bronchiolar epithelial degeneration or necrosis, vascular endothelial degeneration or necrosis, alveolar/interstitial inflammation, bronchial or bronchiolar inflammation, and perivascular inflammation. Lung pathology was scored as follows: 0 (normal), 1 (minimal pathology), 2 (mild pathology), 3 (moderate pathology), or 4 (marked pathology). Total histopathological scores were calculated for individual mice by summing the individual histopathological scores. Mean total histopathological scores were calculated for each experimental group of mice. In cases of disparate scores, the specimen was re-scored by the first pathologist.

### Physical association between S-910823 and A-910823 during intramuscular dosing

To evaluate the physical association between S-910823 and A-910823 during vaccination, mice were intramuscularly administered separate doses of 1 μg S-910823 (30 μL) and 100% (v/v) A-910823 (30 μL) at 0 h, 1 h, or 24 h intervals or a single dose of 1 μg of S-910823 with or without A-910823 (60 μL). Each treatment was performed on Day 0 and Day 14. Whole blood was obtained from each mouse 7 d post-second vaccine administration. Serum samples were prepared from the blood samples on the day of the blood collection and stored at − 80 °C until analysis of neutralizing antibody titers.

### Inflammatory cells and GC reaction in the draining lymph nodes

Mice were intramuscularly administered 1 μg of S-910823 with or without A-910823 on Day 0 and Day 14. To analyze the inflammatory cells, the draining lymph nodes were collected at 3 h and 24 h post-first immunization, and single-cell suspensions were prepared. The lymph node cells were washed and incubated with anti-CD16/32 antibodies (clone 2.4G2, BD Pharmingen, San Diego, CA, USA) and stained for viability using Fixable Viability Dye eFluor 780 (Thermo Fisher Scientific). To identify monocytes, dendritic cells, neutrophils, or eosinophils, the samples were immunostained with fluorescein isothiocyanate (FITC)-conjugated anti-CD11b (clone M1/70, BioLegend, San Diego, CA, USA), phycoerythrin (PE)-conjugated anti-CD19 (clone 6D5, BioLegend), PE-Cy7-conjugated anti-Ly-6C (clone HK1.4, BioLegend), allophycocyanin (APC)-conjugated anti-Ly-6G (clone 1A8, BioLegend), Brilliant Violet (BV)421-conjugated anti-CD11c (clone N418, BioLegend), BV650-conjugated anti-Siglec-F (clone E50-2440, BD Biosciences), and BV785-conjugated anti-MHC class II I-A/I-E (clone M5/114.15, BioLegend) antibodies. Draining lymph nodes were collected on days 1, 7, 14, 17, and 28 post-first immunization to analyze the Tfh cells and GCBs. The lymph node cells were washed and incubated with anti-CD16/32 (clone 2.4G2), stained with the Fixable Viability Dye eFluor 780, and immunostained with FITC-conjugated anti-CD95 (clone SA367H8, BioLegend), PE-conjugated anti-CD19 (clone 6D5, BioLegend), PE-Cy7-conjugated anti-Pdcd1 (clone J43, Thermo Fisher Scientific), Alexa Fluor 647-conjugated anti-GL7 (clone GL7, BioLegend), BV421-conjugated anti-Cxcr5 (clone L138D7, BioLegend), BV510-conjugated anti-Tcrb (clone H57-597, BD Biosciences), and BV650-conjugated anti-CD4 (clone RM4-5, BioLegend) antibodies. The cells were examined using the Novocyte Advanteon Flow Cytometer and the data analyzed using NovoExpress 1.4.0 software.

### Measurement of CXCL13 in the serum

The serum was prepared from the blood samples collected from vaccinated mice on days 1, 7, 14, 15, 17, and 28 post-first administration of S-910823 with or without A-910823. The concentrations of CXCL13 in the serum samples were measured using an AlphaLISA Detection Kit (PerkinElmer), following the manufacturer’s instructions.

### Expression analysis of humoral immune response-related genes in draining lymph nodes

Gene expression was evaluated using mRNA isolated from draining lymph nodes of vaccinated mice on days 1, 7, 14, 17, and 28 post-administration of S-910823 with or without A-910823. Total RNA was extracted from the lymph nodes using QIAzol and a RNeasy Micro Kit (QIAGEN), following the manufacturer’s instructions. The mRNA expression levels of *Aicda*, *Il21*, *Prdm1*, *Il4*, and *Tnfrsf17* were quantified using a One Step PrimeScript RT-PCR Kit (Perfect Real Time) (Takara Bio Inc.) and a 7500 Fast Real Time PCR System (Applied Biosystems, Waltham, MA, USA). The relative expression levels were analyzed using the ΔΔCt method. The expression levels of the target genes were normalized to those of the reference gene *Ubc*.

### Measurement of antigen-specific memory B-cells, memory T-cells, and ASCs

Mice were intramuscularly administered 1 μg of S-910823 with or without A-910823 on Day 0 and Day 14. On Day 83, the mice were sacrificed under anesthesia, and their spleens were collected. Splenocytes were prepared to evaluate antigen-specific memory B-cells and memory T-cells. To detect SARS-CoV-2 spike-protein RBD-specific B-cells, RBD probe proteins were prepared. Briefly, biotinylated SARS-CoV-2 S protein RBD (ACROBiosystems) was pre-incubated with FITC-streptavidin (BioLegend) or BV785-streptavidin (BioLegend) for 30 min. The splenocytes were incubated with anti-mouse CD16/CD32 antibodies (clone 2.4G2, BioXcell) and stained for viability with Zombie NIR dye (BioLegend). The cells were then washed with autoMACS Running Buffer (Miltenyi) and incubated for cell-surface staining with anti-mouse CD19 (clone 6D5, BioLegend), anti-mouse TCR-β (clone H57-597, BD Biosciences), and anti-mouse Nt5e antibodies (clone TY/11.8, BioLegend), and RBD probe proteins for 30 min. Next, the cells were washed and resuspended in autoMACS Running Buffer and analyzed using the Novocyte Advanteon Flow Cytometer. To detect SARS-CoV-2 spike-protein antigen-specific T-cell responses, splenocytes were cultured in 96-well plates with or without SARS-CoV-2-derived peptides (PepTivator SARS-CoV-2 Prot_S1, Prot_S, and Prot_S + , Miltenyi) at a final concentration for each peptide of approximately 1 μg/mL at 37 ℃ for 16 h. Next, the splenocytes were incubated with a Protein Transport Inhibitor Cocktail (Thermo Fisher Scientific) for 6 h, washed with PBS, incubated with anti-CD16/32 antibodies, and stained with Zombie NIR dye. The cells were then immunostained for surface markers using anti-mouse CD4 (clone RM4-5, BD Biosciences), anti-mouse CD8a (clone 53–6.7, BioLegend), and anti-mouse/human CD44 antibodies (clone IM7, BioLegend). The immunostained cells were washed and resuspended in BD Cytofix/Cytoperm Fixation and Permeabilization Solution (BD Biosciences). The cells were then incubated with anti-mouse IFN-γ (clone XMG1.2, Tonbo Biosciences, San Diego, CA, USA), anti-mouse IL-2 (clone JES6-5H4, BioLegend), anti-mouse TNF-α (clone MP6-XT22, BioLegend), anti-mouse IL-4 (clone 11B11, BD Biosciences), and anti-mouse/human IL-5 antibodies (clone TRFK5, BioLegend) for intracellular staining. The cells were analyzed using flow cytometry. For antigen-specific B-cell ELISpot assays, bone marrow cells were collected on Day 83 post-first vaccine administration. SARS-CoV-2 spike-protein-specific ASCs were detected using a B-cell ELISPOT assay with ELISPOT polyvinyl difluoride plates and a mouse IgG ELISpot BASIC Kit (Mabtech Inc., Cincinnati, OH, USA), following the manufacturer’s instructions with some modifications. Briefly, the wells of a 96-well plate were pretreated with 35% ethanol and washed with sterile water, followed by coating with 5 μg/well SARS-CoV-2 S protein (Acro BIOSYSTEMS). The samples were incubated overnight at 4 °C. Next, the samples were washed with PBS and blocked with complete RPMI-1640 medium for 2 h at 37 °C. The bone marrow cells were seeded into the wells in duplicate (1 × 10^6^ cells/well) and incubated for 2.5 h at 37 °C with 5% CO_2_. The samples were washed with PBS-T (Promega) and the secreted antibodies were detected by incubating with biotinylated polyclonal goat anti-mouse IgG (0.1 μg/well) diluted in 0.5% FBS/PBS for 2 h at room temperature. After washing, the samples were incubated with ALP-conjugated streptavidin (Mabtech) diluted in 0.5% FBS/PBS (1:1000) for 1 h at room temperature. The samples were washed and incubated with the substrate BCIP/NBT (Mabtech) (100 μL/well) for 5 min at room temperature. The average number of SFCs per 10^6^ cells was counted for duplicate wells using an immunospot analyzer (Cellular Technology Limited, Cleveland, OH, USA).

### Statistical analysis

All statistical analyses were performed using GraphPad Prism (GraphPad Software Inc., San Diego, CA, USA). Bodyweight and survival curves were analyzed using Dunnett’s multiple comparison and log-rank tests, respectively. Neutralizing antibody titers and eosinophil counts were analyzed using nonparametric tests, followed by the Kruskal–Wallis test. Virus titers were analyzed using Tukey’s multiple comparison test. Cytokine and chemokine levels were analyzed using two-way analysis of variance, followed by Dunnett’s multiple comparison test.

### Reporting summary

Further information on the research design is available in the Nature Research Reporting Summary linked to this article.

### Supplementary Information

Supplementary Information.

## Data Availability

The datasets generated and/or analyzed during the current study are not publicly available due to ethical and privacy concerns. However, they may be made available from the corresponding author upon reasonable request.
