# Author Correction: Immune response and protective efficacy of the SARS-CoV-2 recombinant spike protein vaccine S-268019-b in mice

**DOI:** 10.1038/s41598-024-52772-3

**Published:** 2024-01-31

**Authors:** Tomoyuki Homma, Noriyo Nagata, Masayuki Hashimoto, Naoko Iwata-Yoshikawa, Naomi M. Seki, Nozomi Shiwa-Sudo, Akira Ainai, Keiji Dohi, Eiji Nikaido, Akiko Mukai, Yuuta Ukai, Takayuki Nakagawa, Yusuke Shimo, Hiroki Maeda, Seiki Shirai, Miwa Aoki, Takuhiro Sonoyama, Mamoru Sato, Masataka Fumoto, Morio Nagira, Fumihisa Nakata, Takao Hashiguchi, Tadaki Suzuki, Shinya Omoto, Hideki Hasegawa

**Affiliations:** 1 Laboratory for Bio-Drug Discovery, Shionogi & Co., Ltd., 3-1-1, Futaba-cho, Toyonaka, Osaka 561-0825 Japan; 2 Department of Pathology, National Institute of Infectious Diseases, 4-7-1, Gakuen, Musashimurayama-shi, Tokyo 208-0011 Japan; 3 Laboratory for Bio-Modality Research, Shionogi & Co., Ltd., 3-1-1, Futaba-cho, Toyonaka, Osaka 561-0825 Japan; 4 UMN Pharma Inc., 7F, Tekko Building, 1-8-2, Marunouchi, Chiyoda-ku, Tokyo 100-0005 Japan; 5 Laboratory for Drug Discovery and Disease Research, Shionogi & Co., Ltd., 3-1-1, Futaba-cho, Toyonaka, Osaka 561-0825 Japan; 6 Medical Science Department, Shionogi & Co., Ltd., 8F, Nissei East Building, 3-3-16, Imabashi, Chuo-ku, Osaka 541-0032 Japan; 7 Laboratory of Medical Virology, Institute for Life and Medical Sciences, Kyoto University, 53 Shogoin Kawahara-cho, Sakyo-ku, Kyoto 606-8507 Japan; 8 Department of Virology, Faculty of Medicine, Kyushu University, 3-1-1 Maidashi, Higashi-ku, Fukuoka 812-8582 Japan; 9 Department of Pathology, National Institute of Infectious Diseases, 1-23-1, Toyama, Shinjuku-ku, Tokyo 162-8640 Japan; 10 Center for Influenza and Respiratory Virus Research, National Institute of Infectious Diseases, 4-7-1, Gakuen, Musashimurayama-shi, Tokyo 208-0011 Japan

Correction to: *Scientific Reports* 10.1038/s41598-022-25418-5, published online 02 December 2022

The original version of this Article contained an error in Figure 6 where panel (e) was omitted. The original Figure 6 and accompanying legend appear below.

**Figure 6:**
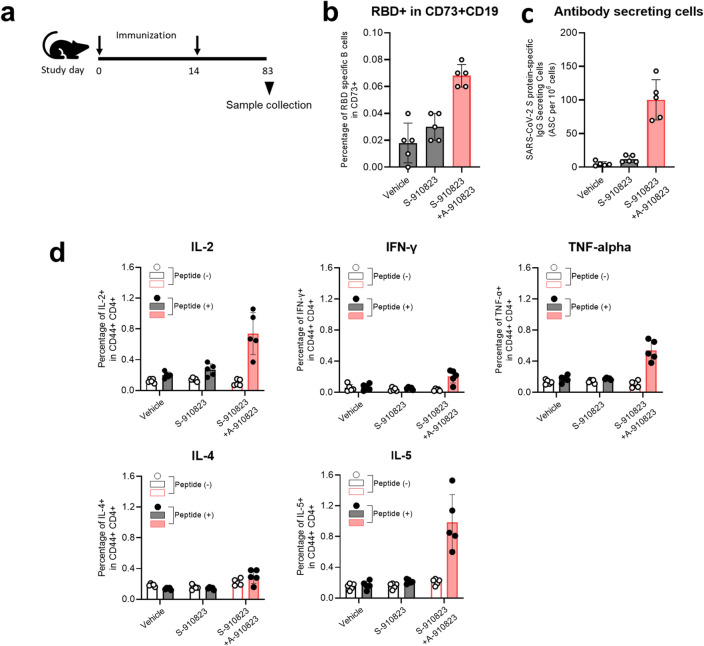
Induction of long-term immunity in mice administered S-910823 plus A-910823. (**a**) Study schedule schematic. On Day 0 and Day 14, female mice were intramuscularly administered 1 μg of S-910823 with or without A-910823 (n = 5/group). Spleens and bone marrow were collected on Day 83. (**b**) Flow cytometric analysis of the percentage of receptor-binding domain (RBD)-specific B-cells among CD73^+^CD19^+^ B-cells. Each circle represents the percentage of cells in an individual mouse. The bars represent mean values, while error bars indicate standard deviation. (**c**) Detection of vaccine-induced antibody-secreting cells (ASCs) in bone marrow. Bone marrow cells were cultured for 2.5 h in an enzyme-linked immunospot (ELISPOT) assay plate wells which had been coated with SARS-CoV-2 spike protein. Results indicate the number of antigen-specific ASCs among 10^6^ bone marrow cells. (**d**, **e**) Percentage of cytokine-producing T cells in the spleen. Splenocytes were restimulated ex vivo with or without SARS-CoV-2 spike-protein-derived peptides for 16 h. Levels of IL-2, IFN-γ, TNF-α, IL-4, and IL-5 from CD44^+^CD4^+^ T cells (**d**) and levels of IFN-γ from CD44^+^CD8^+^ T cells (**e**) were evaluated using cell-surface and intracellular staining and examined using flow cytometry. The open bars indicate non-stimulated splenocytes [peptide (−)], while the solid bars indicate splenocytes stimulated with SARS-CoV-2 spike protein peptides [peptide (+)]. The gray bars represent mice administered vehicle or S-910823 without adjuvant A-910823, while the rose bars represent mice administered S-910823 with A-910823. The open and solid circles represent the results of individual mice. The error bars indicate the standard deviation.

The original Article has been corrected.

