# Supplementary material for: Immune response and protective efficacy of the SARS-CoV-2 recombinant spike protein vaccine S-268019-b in mice
Source: Sci Rep. 2022 Dec 2;12:20861. doi: 10.1038/s41598-022-25418-5 (PMC9718471; doi:10.1038/s41598-022-25418-5)
Supplement: Supplementary file 1 — Supplementary Information. [file 41598_2022_25418_MOESM1_ESM.docx]

**Immune response and protective efficacy of the SARS-CoV-2 recombinant spike protein vaccine S-268019-b in mice**

Tomoyuki Homma, Noriyo Nagata, Masayuki Hashimoto, Naoko Iwata-Yoshikawa, Naomi M. Seki, Nozomi Shiwa-Sudo, Akira Ainai, Keiji Dohi, Eiji Nikaido, Akiko Mukai, Yuuta Ukai, Takayuki Nakagawa, Yusuke Shimo, Hiroki Maeda, Seiki Shirai, Miwa Aoki, Takuhiro Sonoyama, Mamoru Sato, Masataka Fumoto, Morio Nagira, Fumihisa Nakata, Takao Hashiguchi, Tadaki Suzuki, Shinya Omoto, Hideki Hasegawa

**Supplementary Information**


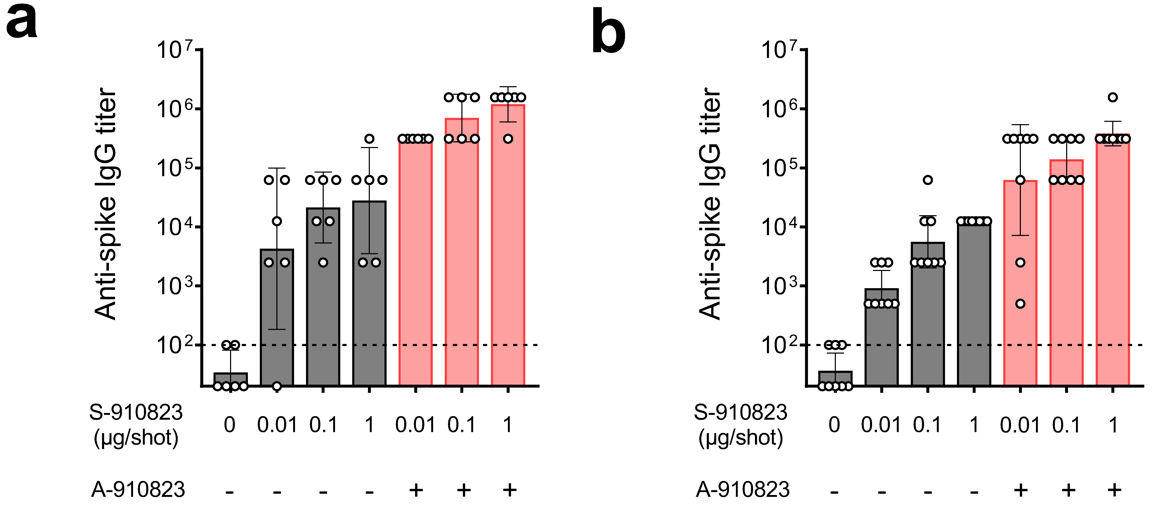


**Fig. S1 Evaluation of anti-spike protein IgG titers in a mouse infection model.** Mice were intramuscularly administered twice with 0.01, 0.1, or 1 µg of S-910823 with or without A-910823 at a 14-day interval. At 20 d or 21 d post-second immunization (Day 34 or Day 35, respectively), the mice were intranasally infected with 2.3 × 10^4^ median tissue culture infectious dose (TCID_50_) of severe acute respiratory syndrome coronavirus 2 (SARS-CoV-2), (**a**) mouse-adapted strain QHmusX (n = 6/group), or (**b**) Beta strain (TY-8-612) (n = 8/group). The serum samples were prepared from blood samples collected from the mice on day 14 post-second immunization. Anti-spike protein IgG titers are defined as the reciprocal of the highest dilution whose value was greater than the cut-off value (mean value of dilution buffer + 0.1). When the value was less than the cut-off value of the first dilution, the titer was defined as 20 for the first dilution factor. The bars represent the geometric mean IgG titers (error bars indicate a 95% confidence interval). The circles represent the titers in individual mice. The dotted lines represent the lower limit of detection.

**
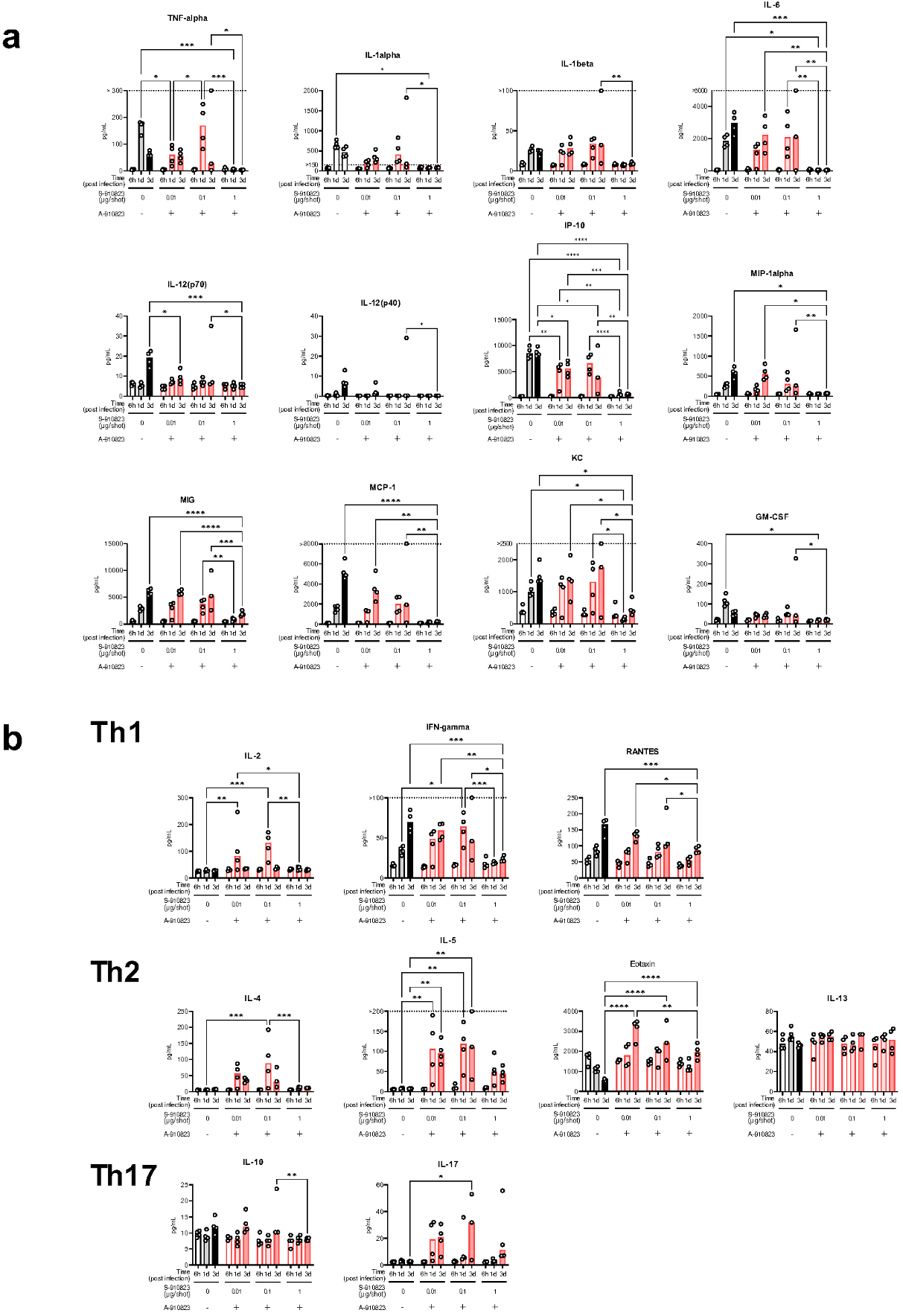
**

**Fig. S2 Inflammatory cytokine and chemokine levels in mouse lungs after severe acute respiratory syndrome coronavirus 2 (SARS-CoV-2) challenge infection.** Female mice were intramuscularly immunized twice with S-268019-b containing various amounts of S-910823 antigen with or without adjuvant A-910823 at a 14-day interval. The study schedule details are provided in Fig. 2a of the main manuscript. Lung samples were collected from the mice at 6 h, 1 d, and 3 d post-infection with the mouse-adapted SARS-CoV-2 D614G strain QHmusX and homogenized. The cytokine and chemokine levels were measured using a commercial mouse cytokine-chemokine magnetic bead panel 96-well plate assay kit. (**a)** Inflammatory cytokines/chemokines. (**b)** Th1-associated, Th2-associated, and Th17-associated cytokines/chemokines. The bars represent median concentrations. The circles represent the values of individual mice. Statistical significance was determined using two-way analysis of variance multiple comparison tests (**P* < 0.05, ***P* < 0.01, ****P* < 0.001, and *****P* < 0.0001).


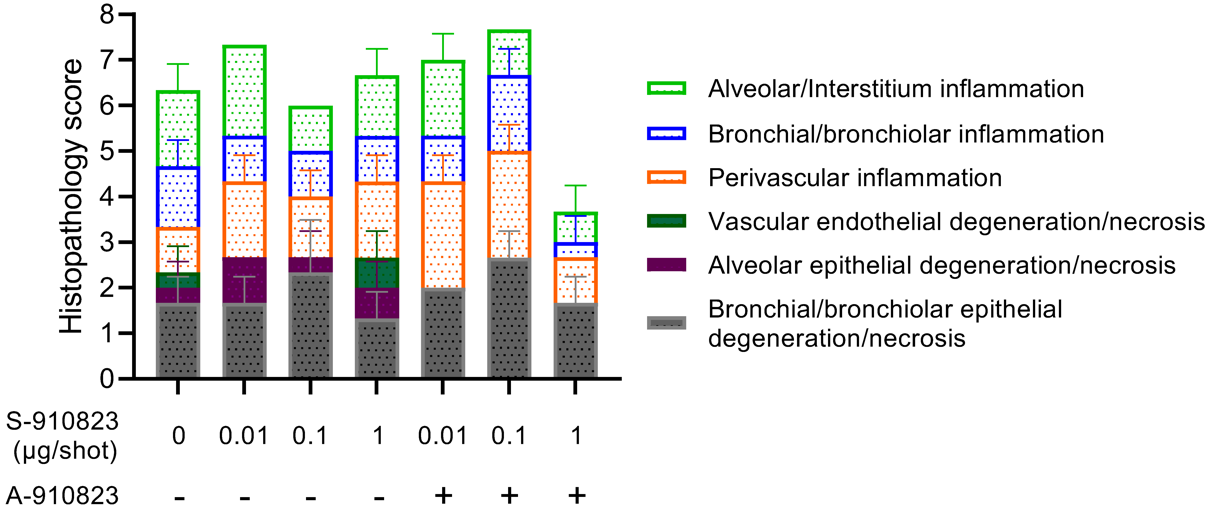


**Fig. S3 Lung histopathology scores in mice subjected to a severe acute respiratory syndrome coronavirus 2 (SARS-CoV-2) Beta variant challenge.** Female mice were intramuscularly immunized twice with S-268019-b containing various amounts of S-910823 antigen with or without adjuvant A-910823 at a 14-day interval. The study schedule details are provided in Fig. 2a of the main manuscript. Lung histopathology scores were evaluated in three of the eight mice in each group. The lung tissues were collected on day 10 post-challenge from mice challenge infected with the SARS-CoV-2 Beta variant.
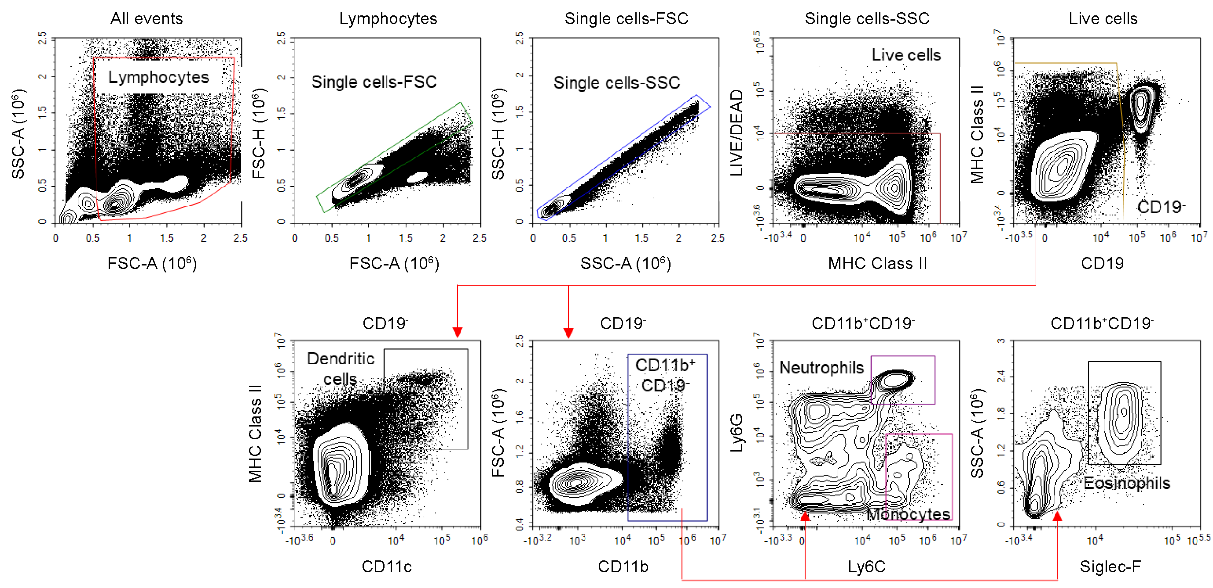


**Fig. S4 Representative scatter plots and gating strategy for the quantification of the myeloid cell population.** Examples of flow cytometry contour plots demonstrating the gating strategy used to identify dendritic cells, monocytes, neutrophils, and eosinophils populations in the draining lymph nodes of mice immunized with S-910823 plus A-910823.


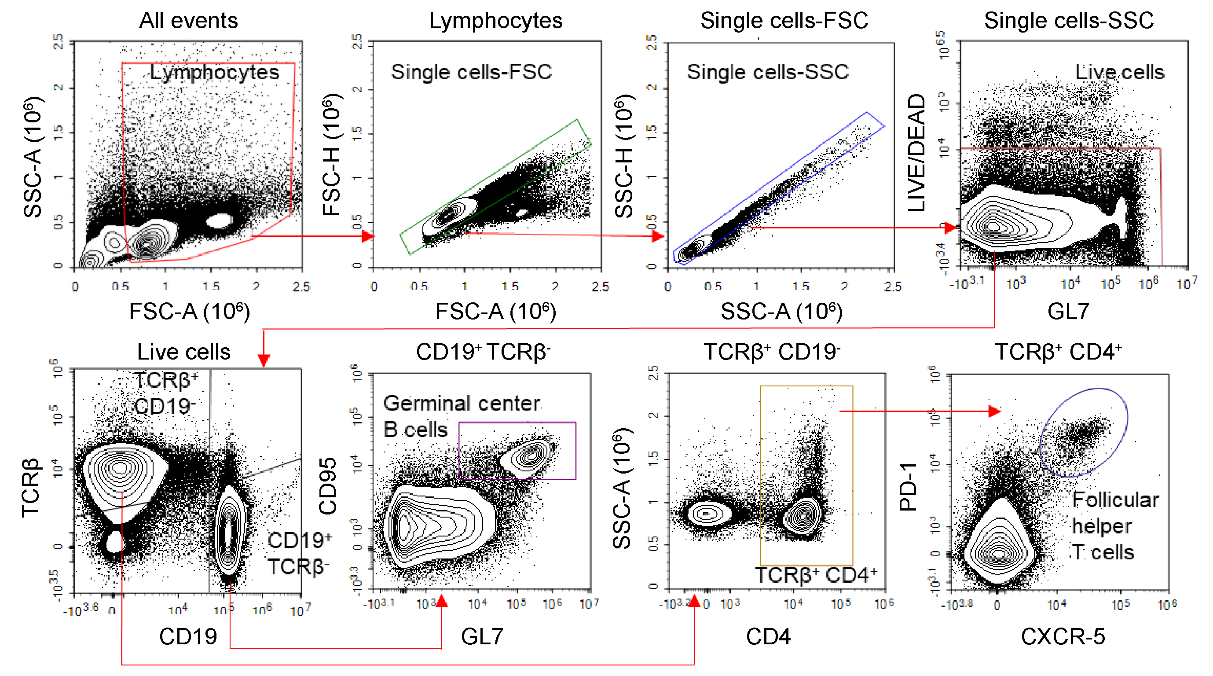


**Fig. S5 Representative scatter plots and gating strategy for the quantification of follicular helper T cells (Tfh cells) and germinal center B cells (GCBs).** Examples of flow cytometry contour plots demonstrating the gating strategy used to identify Tfh and GCB populations from the draining lymph nodes of mice immunized with S-910823 plus A-910823.

**
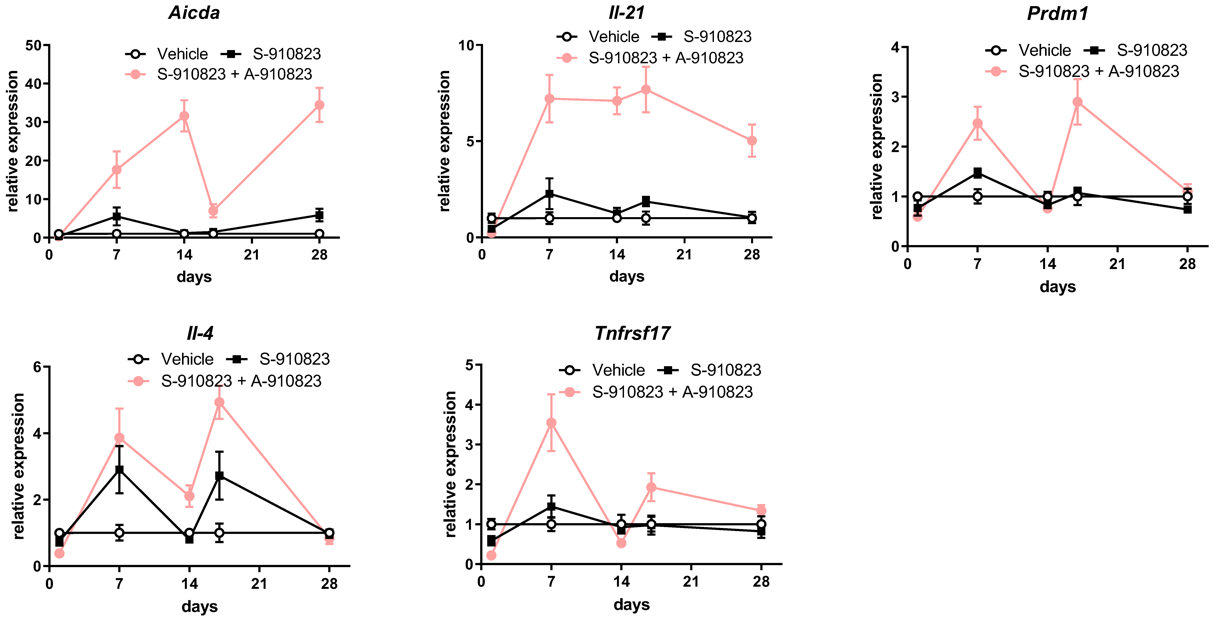
**

**Fig. S6 Expression levels of genes associated with humoral responses in draining lymph nodes of mice immunized with S-910823 plus A-910823.** Female mice were twice intramuscularly administered vector or immunized with S-910823 with or without A-910823 at a 14-day interval. On days 1, 7, 14, 17, and 28 post-first immunization, mRNA levels of target genes in the draining lymph nodes were determined using quantitative real-time polymerase chain reaction analysis (n = 5 mice/time point). The expression levels of genes related to germinal center formation and B-cell maturation (*Aicda*, *Il21*, *Prdm1*, *Il4*, and *Tnfrsf17*) were measured. The relative expression levels were analyzed using the ΔCt method. The expression levels of target genes were normalized to those of *Ubc*. The plots represent the mean relative expression of each gene (error bars indicate a 95% confidence interval).

**
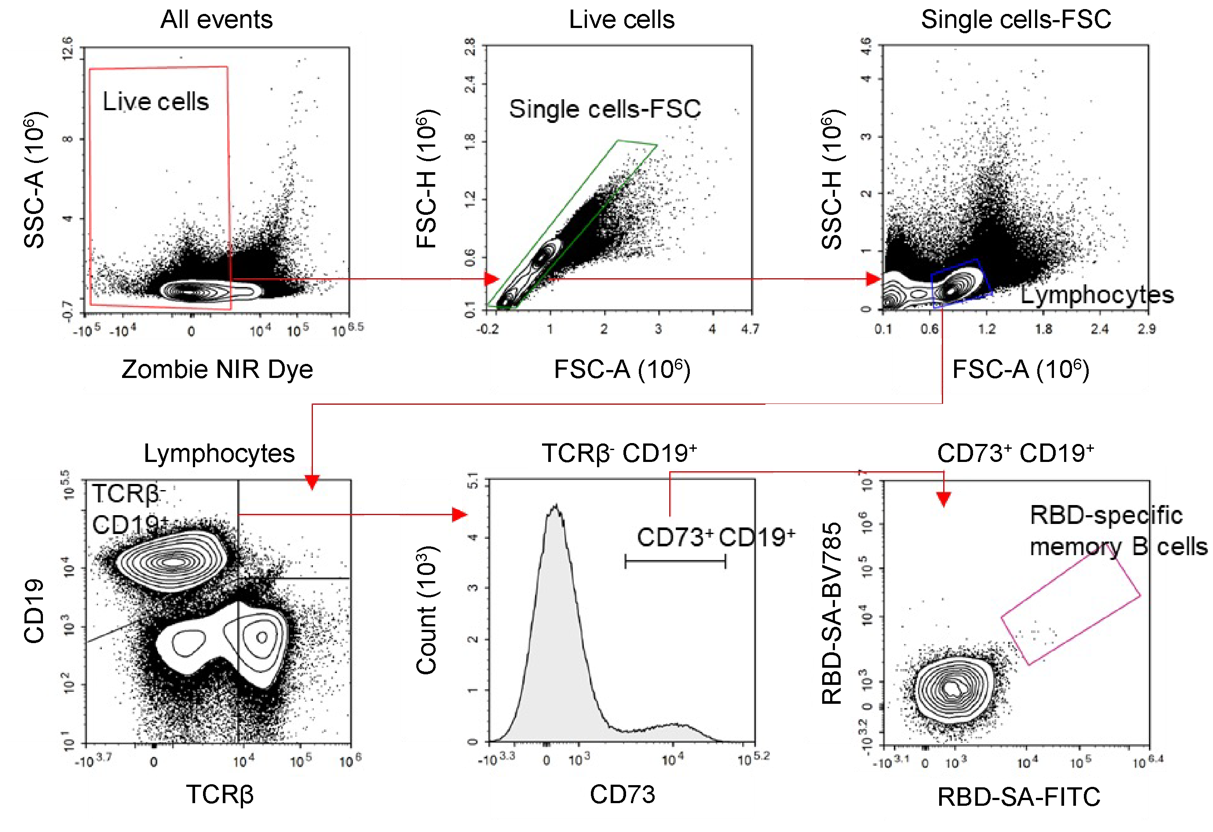
**

**Fig. S7 Gating strategy for the quantification of memory B-cells.** Splenocytes were stained and analyzed using flow cytometry. B-cells were defined as TCRβ^−^ and CD19^+^. Receptor binding domain (RBD)-specific memory B-cells were defined as the RBD-SA-BV785 and RBD-SA-fluorescent isothiocyanate (FITC) double-positive fraction among CD73^+^CD19^+^ B cells.


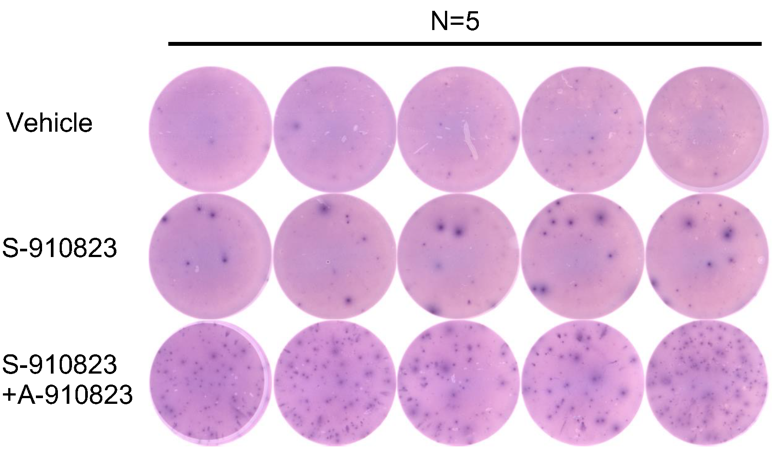


**Fig. S8 Representative images of enzyme-linked immunospot assay plates used to detect antibody-secreting cells.** Each spot indicates one cell that secreted the anti-spike IgG. The assay was performed using duplicate samples.

**
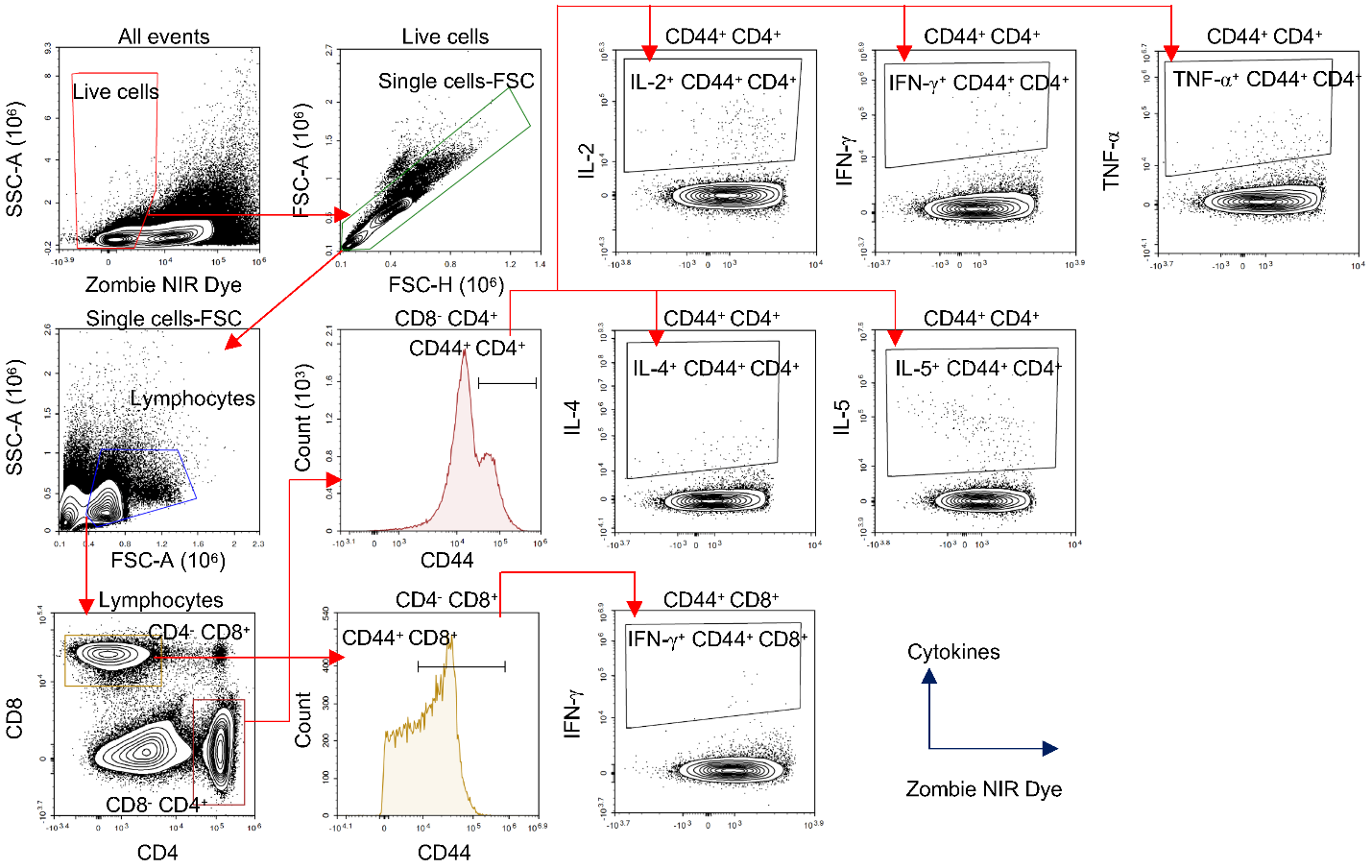
**

**Fig. S9 Gating strategy for the quantification of memory T cells.** Splenocytes restimulated *ex vivo* were stained for cell surface and intracellular markers. Lymphocytes were gated on CD4^+^ T cells and CD8^+^ T cells. IL-2, IFN-γ, TNF-α, IL-4, and IL-5 from CD44^+^CD4^+^ T cells and Ifng from CD44^+^CdD8^+^ T cells were gated.


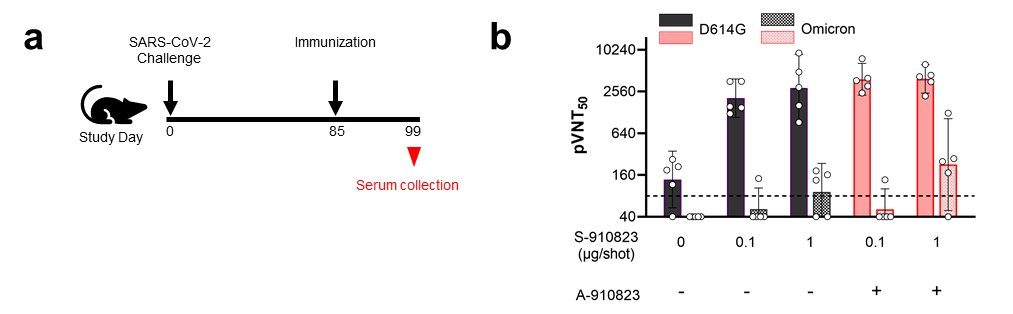


**Fig. S10 Boosting administration of S-910823 plus A-910823 after severe acute respiratory syndrome coronavirus 2 (SARS-CoV-2) infection in mice.** (**a**) Study schedule schematic. On 85 d post intranasally infection with severe acute respiratory syndrome coronavirus 2 (SARS-CoV-2) MA-P10, mice were intramuscularly administrated with S-910823 plus A-910823. After 14 d post-immunization, serum samples were collected and subjected to neutralizing antibody titer testing. (**b**) Neutralizing antibody titer in the serum against SARS-CoV-2 spike pseudotyped lentivirus 14 d after immunization. Each bar represents the geometric mean titer, and error bars indicate a 95% confidential interval. Each circle represents the neutralizing antibody titer in individual mice. The dotted line indicates the lower limit of detection.


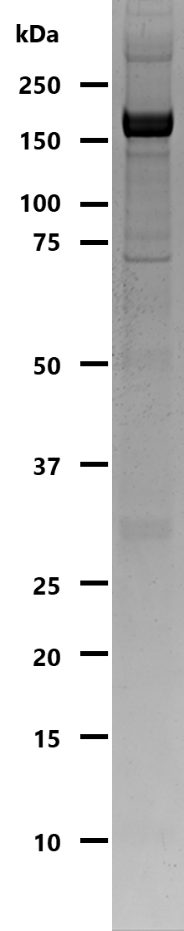


**Fig. S11 Analysis of purified baculovirus-expressed S-910823 antigen by SDS-PAGE and Coomassie Brilliant Blue staining.** The recombinant full-length spike protein S-910823 was expressed in baculovirus-infected cells and extracted, followed by filtration, purification, and buffer exchange. The purified S-910823 was analyzed by SDS-PAGE under reducing condition and Coomassie Brilliant Blue staining.
